# Intracellular STING inactivation sensitizes breast cancer cells to genotoxic agents

**DOI:** 10.18632/oncotarget.12858

**Published:** 2016-10-24

**Authors:** Julie Gaston, Laura Cheradame, Vanessa Yvonnet, Olivier Deas, Marie-France Poupon, Jean-Gabriel Judde, Stefano Cairo, Vincent Goffin

**Affiliations:** ^1^ Inserm, U1151, Institut Necker Enfants Malades (INEM), University Paris Descartes, Faculty of Medicine, Paris, France; ^2^ XenTech, 4 rue Pierre Fontaine, 91000 Evry, France; ^3^ LTTA Center, Department of Morphology, Surgery and Experimental Medicine, University of Ferrara, Italy

**Keywords:** interferon, STAT1, PARP12, recurrence

## Abstract

Activation of the IFN/STAT1 pathway is closely associated with drug response and recurrence of breast cancer treated by chemotherapy. The aim of the current study was to elucidate the molecular mechanisms involved upstream and downstream of this pathway in order to identify distinct entities that might be manipulated to improve treatment efficacy. Four breast cancer cell lines (T-47D, MCF7, MDA-MB-231 and HBCx-19 established from the eponymous PDX) were treated *in vitro* with mafosfamide, a DNA damage inducer. In two of these cell lines (MCF7 and HBCx-19), genotoxic treatment upregulated type I IFN expression leading to paracrine activation of IFN/STAT1 signaling pathway after 6–8 days. We show that STING, a well-characterized inducer of IFN in immune cells, is rapidly triggered in MCF7 cells under genotoxic stress and forms nuclear foci that co-localize with phosphorylated IRF-3 and γH2AX. STING silencing abrogated chemotherapy-induced type I IFN production and signaling and potentiated genotoxic treatment efficacy as it promoted cell death extent and delayed cell colony regrowth. Similar results were obtained after silencing PARP12, one selected gene of the IFN/STAT1 pathway fingerprint. In summary, this study provides the first demonstration of STING activation in breast cancer cells. Our data suggest that genotoxic-induced, STING-mediated type I IFN signaling is a cell-intrinsic mechanism of breast cancer cell survival and regrowth.

## INTRODUCTION

Chemotherapy is active in numerous cancers, reducing tumor growth and lengthening patient survival. However these benefits are not constant and frequently transient, reflecting the ability of cancer cells to survive to drug toxicity. Resistance to chemotherapy can be observed *de novo*, as identified by the lack of tumor response. Escape to treatment can occur after an initial response, defining a process of drug-resistance differing from the *de novo* resistance. Such an adaptive survival is frequent and responsible for tumor recurrences after response to chemotherapy. Thus, improving the efficacy of treatment by prevention of cancer cell survival and recurrence is currently an active area of research [1] and of rational hope.

Using several breast cancer patient-derived xenografts (PDXs), we were recently able to distinguish xenografts which resisted *de novo* to chemotherapy, and others which initially regressed under treatment, but progressed with constant recurrences [2]. Furthermore, the responses to chemotherapy were tightly related to the activation of IFN/STAT1 signaling in post-treatment residual cancer cells [2]. Indeed, the upregulation of an IFN fingerprint covering 140 IFN-stimulated genes (ISGs) was observed in responding tumors only [2]. This IFN-related response was correlated with STAT1 phosphorylation and massive DNA damage, ɣH2AX. However, neither the actual mechanisms by which chemotherapy triggered the IFN/STAT1 pathway in these breast cancer PDXs nor the actual contribution of the ISG fingerprint to the tumor response were elucidated. Using human-specific molecular tools, both type I (α, β) and II (ɣ) IFNs were detected in tumor cells in response to treatment. Since breast cancer cells have been shown to express IFN receptors [3], these observations suggested that activation of the IFN/STAT1 pathway might be induced by an autocrine/paracrine mechanism.

Both types I and II IFNs are typical cytokines classically secreted by immune cells to induce immune cell activation and differentiation in response to pathogen aggression [4, 5]. Several studies have shown that the presence of tumor infiltrating immune cells was a factor of favorable prognosis in various human solid tumors [6–9]. The presence of IFNs in the tumor microenvironment has been widely documented [for a review, 10] and they are usually viewed as active contributors to the antitumor processes mediated by the immune system. Furthermore, a recent study suggested that cancer cell-intrinsic type I IFN signaling may contribute to chemosensitization [11]. Otherwise, the transcriptomic profiling of biopsies from women with locally advanced/high risk early stage breast cancers receiving neoadjuvant chemotherapy revealed that increased ISG expression at the time of surgery (compared to pre-treatment levels) was associated with early cancer recurrence [12]. This finding nicely correlated the observation that STAT1, the main downstream signaling target of IFN receptors, was constitutively activated in cancer cells surviving chronic treatments inducing DNA damage [13, 14]. In agreement, an IFN-related DNA damage resistance signature (IRDS) gathering STAT1 and 48 other genes was identified as a predictive marker of recurrence after radiotherapy [15, 16]. Of note, the IRDS signature showed only partial overlap with the IFN/STAT1 fingerprint that we identified in PDXs [Ref. 2 and Table 1]. Taken together, these data underline the functional complexity of IFNs secreted into the tumor microenvironment, which may exert opposite actions on tumor response to treatment depending on the nature of the target cell (immune vs neoplastic) and on signal kinetics (acute vs chronic).

**Table 1:** IFN/STAT1 fingerprint induced in breast cancer PDXs after chemotherapy treatment

	IFN/STAT1 fingerprint (selected 21 gene list)^a^	
*Gene name*	*Full name*	IRDS^b^
**BST2**	bone marrow stromal cell antigen 2	X
**IFI44**	interferon-induced protein 44	X
**IFITM1**	interferon induced transmembrane protein 1 (9-27)	X
**STAT1**	Signal transducer and activator of transcription 1	X
**MX1**	myxovirus (influenza virus) resistance 1, interferon-inducible protein p78 (mouse)	X
**IFIT1**	interferon-induced protein with tetratricopeptide repeats 1	X
**DDX60**	DEAD (Asp-Glu-Ala-Asp) box polypeptide 60	X
**OAS1**	2′,5′-oligoadenylate synthetase 1, 40/46 kDa	X
**OAS2**	2′-5′-oligoadenylate synthetase 2, 69/71 kDa	
**PARP12**	**poly (ADP-ribose) polymerase family, member 12**	
**PARP9**	poly (ADP-ribose) polymerase family, member 9	
**SAMD9L**	sterile alpha motif domain containing 9-like	
**STAT2**	Signal transducer and activator of transcription 2	
**UBE2L3**	ubiquitin-conjugating enzyme E2L 3	
**IFI44L**	interferon-induced protein 44-like	
**IFI6**	interferon, alpha-inducible protein 6	
**IFIT3**	interferon-induced protein with tetratricopeptide repeats 3	
**IRF9**	ring finger protein 31	
**LAMP3**	lysosomal-associated membrane protein 3	
**ZNFX1**	zinc finger, NFX1-type containing 1	
**CLDN1**	Claudin 1	

a Genes selected to represent the diversity of the IFN/STAT1 fingerprint induced in residual tumor cells after chemotherapy in responder PDXs [2].

b Genes of the IFN/STAT1 fingerprint identified as IRDS [15].

In immune cells, it has been shown that type I IFN production and downstream signaling was stimulated by the STING pathway [17]. STING (stimulator of IFN genes), also referred to as nuclear envelope transmembrane protein (NET) 23 or transmembrane protein 173 (TMEM173), is a transmembrane protein reported to be located in the endoplasmic reticulum [18–20]. One mechanism of STING activation has been recently elucidated. In the presence of cytosolic double stranded (ds) or single stranded (ss) DNA originating from pathogens, the cytosolic DNA sensor cyclic GMP-AMP synthase (cGAS) catalyzes the production of 2′3′ cyclic GMP-AMP (cGAMP) which binds to and activates STING [21–23]. Activated STING then relocalizes to perinuclear regions and ultimately contributes to the activation of type I IFN expression by triggering Tank-binding kinase (TBK)-1-mediated phosphorylation of the transcription factor IFN-regulatory factor (IRF)-3, leading to its nuclear translocation [24–26]. As such, the STING/IFN pathway plays a critical role in anti-microbial innate immunity [23, 26, 27]. Based on this evidence, the paradigm emerges that increasing type I IFN expression within the tumor microenvironment may potentiate the antitumor immune response with expected therapeutic benefit [5]. One of the strategies currently under investigation involves the use of STING agonists which have been shown to induce IFN production by tumor-infiltrating lymphocytes (TILs) and in turn to promote rejection of established tumors in immune-competent host mice [28].

In this context, and following up our recent findings using breast cancer PDXs [2], the present study was designed with the double aim to elucidate i) the molecular mechanisms by which cell-intrinsic IFN signaling was triggered in cancer cells in response to chemotherapy, and ii) the ultimate contribution of the ISG fingerprint to the tumor response to treatment. In order to avoid any interference with biological responses induced by the immune cells present in the tumor microenvironment *in vivo*, we privileged an *in vitro* approach. We first identified breast cancer cell lines mimicking *in vitro* the drug-induced activation of the IFN/STAT1 signature observed *in vivo*, then we used these models to decipher both the upstream mechanisms and downstream consequences of this IFN/STAT1 fingerprint. This study provides the first evidence that chemotherapy used at sub-lethal doses can trigger the STING/IFN pathway in some cancer cells, which in turn contributes to survival to treatment via specific IFN/STAT1 target genes. We identified PARP12 as one such active contributor of this pathway involved in post-chemotherapy survival and re-growth of breast cancer cells.

## RESULTS

### Induction and characterization of an IFN/STAT1 fingerprint in cancer cells under genomic stress

**Figure 1:**
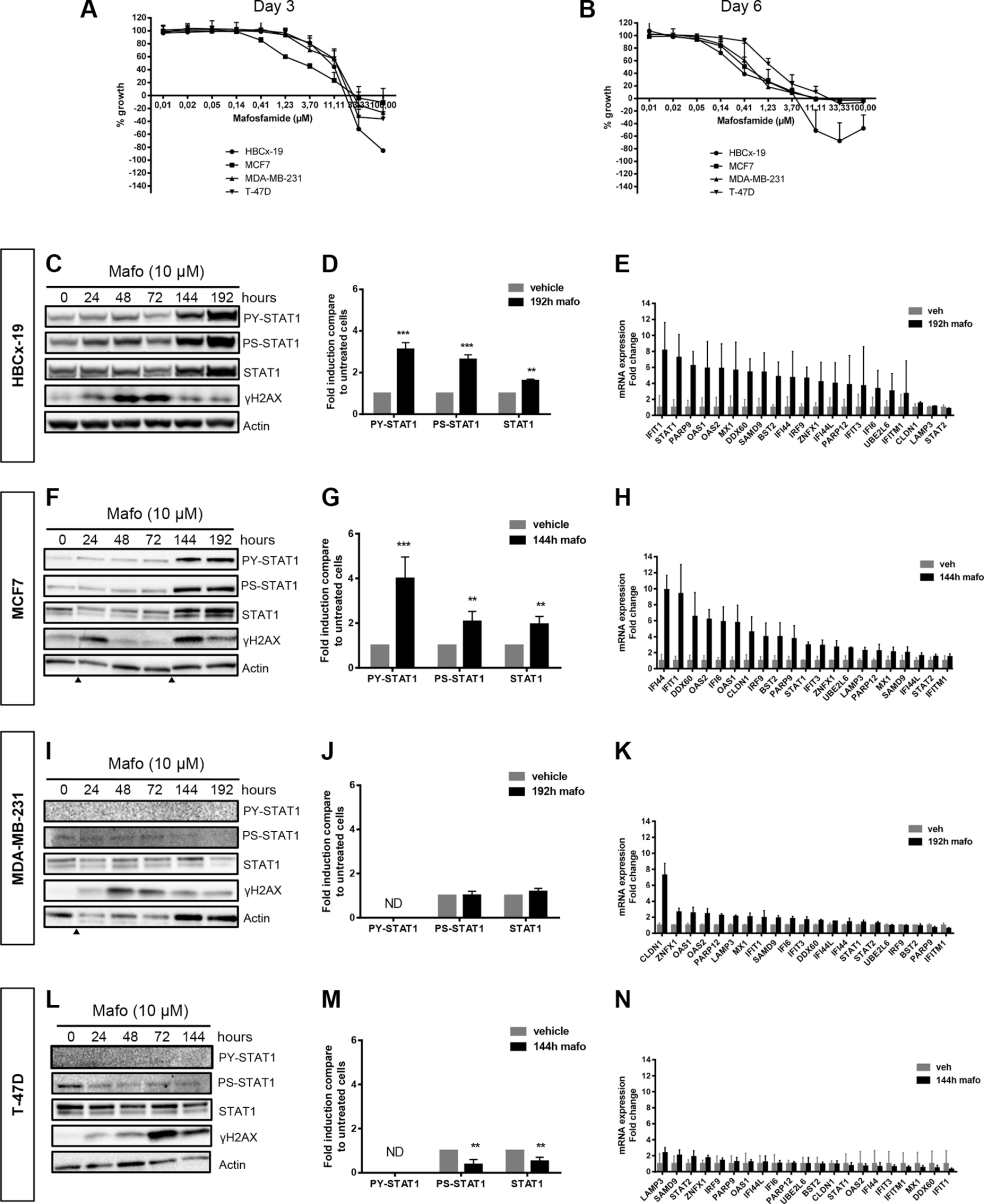
STAT1 phosphorylation and up-regulation of IFN-inducible genes is observed in some but not all breast cancer cells in response to mafosfamide treatment (**A**, **B**) Dose-response effect of mafosfamide on the *in vitro* proliferation of HBCx-19, MCF7, MDA-MB-231 and T-47D cells 3 (A) or 6 (B) days after treatment. Results (*n* = 3 independent experiments) are expressed as percentage of growth with respect to the increased cell density between day 0 and days 3 or 6 in the absence of treatment. (**C**) Western blotting analysis of P-STAT1^Y701^, P-STAT1^S727^ and total STAT1 in HBCx-19 cells showing the time course induction of the STAT1 pathway following mafosfamide treatment (added at T0). ɣH2AX reflects drug-induced DNA damage. (**D**) Quantification of P-STAT1^Y701^, P-STAT1^S727^ and total STAT1 protein levels (normalized to actin levels, mean ± SD) in mafosfamide-treated (192 h) HBCx-19 cells relative to untreated conditions (two-way ANOVA and post-hoc Sidak's multiple comparison test). (**E**) qRT-PCR analysis of the 21 gene signature representative of the IFN/STAT1 fingerprint (Table 1) after 192 h mafosfamide treatment (mean ± SD, *n* = 3 per group compared to cells treated with vehicle). The same analyses as described in C–E were performed for MCF7 cells (**F**–**H**), MDA-MB-231 (**I**–**K**) and T-47D (**L**–**N**) cells. Arrowheads below immunoblots separate noncontiguous parts of the same membrane.

Preliminary screening of various breast cancer cell models identified MCF7, T-47D and MDA-MB-231 cell lines and HBCx-19, a primary cell line derived from the eponymous PDX, as those exhibiting the most robust molecular responses to genotoxic stress (see below). These four cell lines reflect the breast cancer diversity according to their estrogen receptor (ER) status, HER2/Neu expression and p53 mutations (Table 2) [29]. A proliferation test was performed *in vitro* to quantify the effects of mafosfamide, the active metabolite of cyclophosphamide used *in vivo*. We determined that the total growth inhibition (TGI) concentration index, i.e. the dose of drug at which the number of living seeded cells remains unchanged after 3 or 6 days of treatment, was for the four cell lines in the range of 17–18 μM and 4–12 μM, respectively (Figure 1A, 1B and Table 3). As shown in Supplementary Figure S1 using MCF7 cells, 10 μM mafosfamide treatment blocked cells in G2/M (Supplementary Figure S1A, S1B), induced progressive cell death (Supplementary Figure S1C) and concomitantly reduced proliferation as reflected by the decrease of Ki-67 staining (Supplementary Figure S1D, S1E). According to these data, the sub-lethal drug concentration of 10 μM was chosen throughout this study to investigate the consequence of genotoxic stress while maintaining a sufficient number of living cells for performing molecular analyses.

**Table 2:** Characteristics of the four breast cancer cell lines used in this study [see Ref.64]

Cell lines	Origin	ER status	p53 status	Her2 levels
MCF7	ATCC	+	WT	very low
T-47D	ATCC	+	mutated	moderate
MDA-MB-231	ATCC	−	mutated	low
HBCx-19	PDX	+	WT	N.D.

Abbreviations: N.D.: not determined.

**Table 3:** *In vitro* profile of several breast cancer cell lines to mafosfamide sensitivity

	Day 3		Day 6	
	GI_50_ ± SD (μM)	TGI ± SD (μM)	GI_50_± SD (μM)	TGI ± SD (μM)
**MCF7**	2.7 ± 0.7	18.0 ± 6.2	0.6 ± 0.2	11.2 ± 0.9
**T-47D**	12.1 ± 1.5	17.5 ± 1.5	1.5 ± 0.3	12.1 ± 2.2
**MDA-MB-231**	11.4 ± 5.2	17.2 ± 3.1	0.5 ± 0.3	11.0 ± 0.5
**HBCx-19**	10.1 ± 2.8	18.13 ± 1.9	0.6 ± 0.6	4.1 ± 2.2

GI_50_ and TGI index concentrations were calculated 3 or 6 days after mafosfamide treatment (results are mean of at least three independent experiments). GI_50_ was calculated as the drug concentration that reduced the number of cells to 50% of the number of cells before drug addition. TGI is the drug concentration that achieved total growth inhibition of cells.

The status of IFN/STAT1 signaling in response to genotoxic stress was analyzed by western blot. In the four cell lines, we monitored STAT1 phosphorylation on both tyrosine (Y701) and serine (S727) residues and the level of STAT1 expression, since the latter is *per se* a transcriptional target of the IFN/STAT1 pathway [30]. In HBCx-19 cells (Figure 1C, 1D) and MCF7 (Figure 1F, 1G) cells, serine/tyrosine STAT1 phosphorylation and STAT1 protein up-regulation were detected 144 h after addition of mafosfamide, and these effects persisted (MCF7) or even increased (HBCx-19) until 192 h. Using qPCR analysis, we detected an increased expression of the majority of the IFN/STAT1 signature (21 genes) that we previously identified as representative of the drug-induced ISGs in breast cancer PDXs (Figure 1E, 1H) [2]. Based on these observations, HBCx-19 and MCF7 were identified as ‘IFN-responsive’ cell lines, i.e. cells exhibiting STAT1 pathway activation and ISG upregulation in response to genotoxic treatment. In contrast, MDA-MB-231 (Figure 1I–1K) and T-47D (Figure 1L–1N) were identified as ‘IFN-non responsive’ cell lines, as neither STAT1 pathway activation nor ISG upregulation were observed in response to genotoxic stress. The absence of response in IFN-non responsive cells was not due to the lack of drug-induced DNA damage since phosphorylated H2AX (referred to as ɣH2AX) was similarly detected by western blot in the four cell lines (Figure 1C, 1F, 1I, 1L) [31].

Induction of ISGs has been reported to occur during cell senescence [32], therefore we addressed whether the senescence-associated beta-galactosidase (SA-β-gal) could discriminate IFN-responsive *versus* IFN-non responsive cell lines. Using MCF7 (IFN-responsive) and T47D (IFN-non responsive) as representative cell lines this was clearly not the case (Supplementary Figure S2), suggesting that expression of the IFN/STAT1 signature was not linked to cell senescence.

Together these data indicate that *in vitro* and in the absence of immune cells, genotoxic treatment can trigger IFN signaling in some but not all breast cancer cell lines, exactly as observed *in vivo* using PDXs [2].

### Cell-autonomous secretion of type I IFNs is observed in response to genotoxic stress

**Figure 2:**
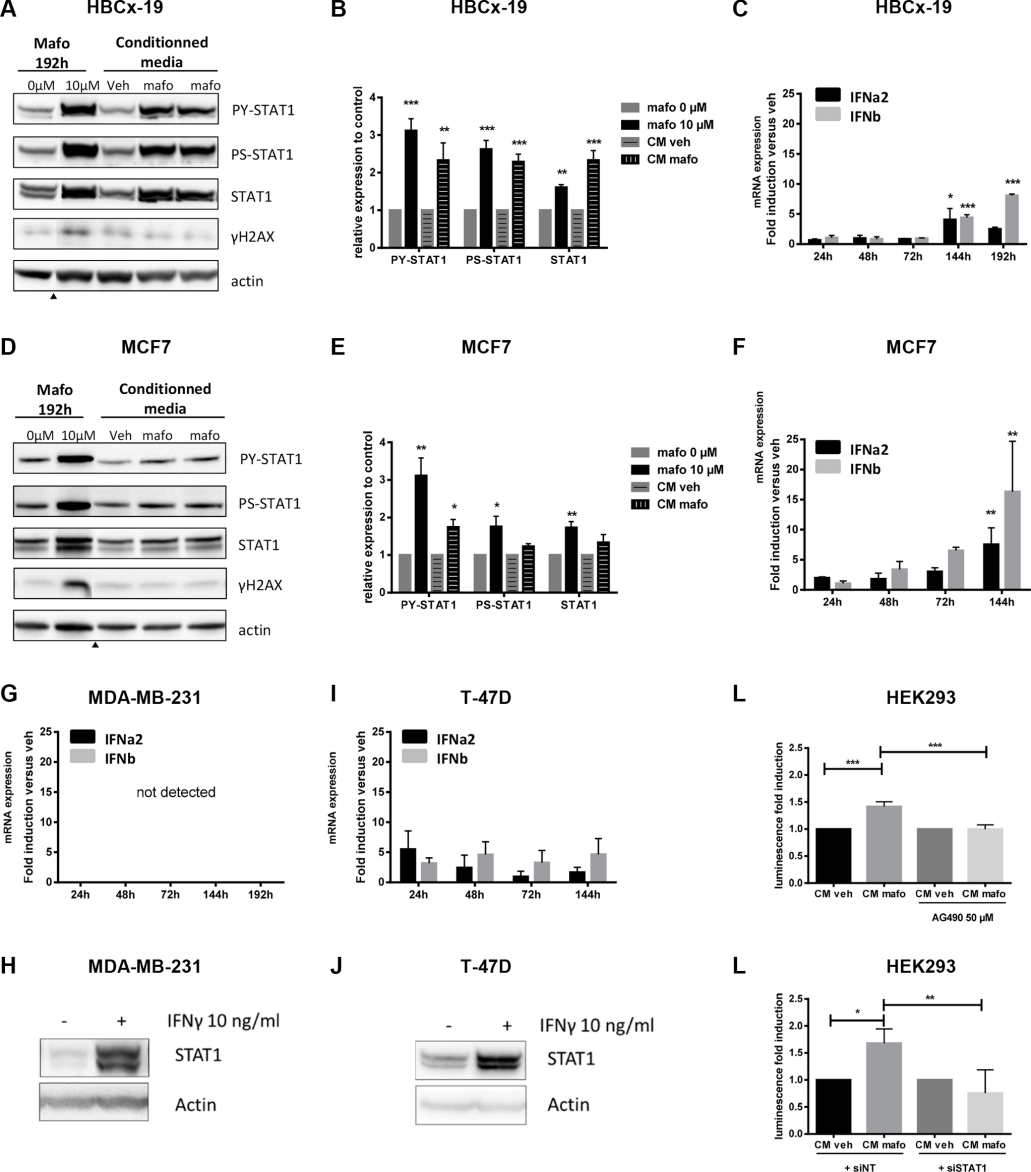
Paracrine IFN signaling is induced by mafosfamide treatment (**A, B, D, E**) HBCx-19 (A, B) and MCF7 (D, E) cells were stimulated for 192 h with mafosfamide *versus* vehicle, or for 24 h with conditioned medium from cells that were stimulated with mafosfamide (CM^mafo^, shown in duplicate) *versus* vehicle (CM^veh^), then P-STAT1^Y701^, P-STAT1^S727^ and total STAT1 were analyzed by western blotting and quantified as described in the legend to Figure 1. Arrowheads below immunoblots separate noncontiguous parts of the same membrane. (**C**, **F**, **G**, **I**) The time-course expression of IFNα2 and IFNβ in HBCx-19 (C), MCF7 (F), MDA-MB-231 (G) and T-47D (**I**) cells treated with mafosfamide versus vehicle was performed by qRT-PCR (*n* = 3, mean ± SD, two-way ANOVA and post-hoc Tukey's multiple comparison test). (**H**, **J**) Western blot of total STAT1 and actin expression in MDA-MB-231 (H) and T47D (J) cells after 72 h stimulation with 10 ng/ml IFNɣ. (**K**, **L**) Activation of ISRE-luciferase reporter plasmid by CM^mafo^ with/without 50 μM AG490 (K) or 20 nM non targeted siRNA (siNT) versus siSTAT1 (L). For each condition the results are expressed relative to CM^veh^ after normalization to Renilla luciferase values (*n* = 3, mean ± SD, two-way ANOVA and post-hoc Sidak's multiple comparison test).

Based on these results, we reasoned that the activation of STAT1 pathway in tumor cells treated with mafosfamide could result from cell-autonomous secretion of cytokines, known to trigger this pathway. We used a conditioned medium approach to address this issue (Supplementary Figure S3). MCF7 and HBCx-19 cells were incubated with mafosfamide (or vehicle) for 6 h, then cells were washed and shifted to fresh, mafosfamide-free, medium. Cell conditioned media (CM^mafo^ or CM^veh^) were collected 144 h (MCF7) or 192 h (HBCx-19) later (i.e. the time to get maximal STAT1 activation, see Figure 1) then added respectively to naive MCF7 or HBCx-19 cells for 24 h. In HBCx-19, CM^mafo^ markedly induced STAT1 tyrosine/serine phosphorylation and total STAT1 up-regulation as efficiently as mafosfamide treatment (Figure 2A, 2B). Similar effects were observed in MCF7 cells albeit with lower efficiency since only upregulation of STAT1 tyrosine phosphorylation achieved significance (Figure 2D, 2E). Together, these results indicate that short time exposure to genotoxic drug is sufficient to induce secretion of ligands able to trigger the STAT1 pathway via a paracrine signaling.

Based on previous reports suggesting that type I and type II IFNs are upregulated in PDXs treated with genotoxics [2, 11], we focused on IFN ligands for further investigations. First, we performed a time-course follow-up of type I and type II IFN mRNA expression in response to mafosfamide treatment. In both IFN-responsive cell lines (HBCx-19 and MCF7), a significant increase in type I IFN expression was observed 144 h/192 h after treatment (Figure 2C, 2F), which matched the detection of the IFN/STAT1 signature at these time points (Figures 1E, H). Type II IFN mRNAs were below the detection threshold in both cell lines (not shown). By contrast, IFN-non responsive cell lines (MDA-MB-231 and T-47D) failed to show any significant upregulation overtime of type I or type II IFN expression in response to mafosfamide treatment (Figure 2G, 2I). Since activation of STAT1 pathway could be observed when these cells were stimulated with purified IFNs (Figure 2H, 2J), this strongly suggests that the absence of IFN/STAT1 signaling in response to mafosfamide treatment in IFN-non responsive models (Figure 1I–1N) is linked to the lack of IFN escalation in these experimental conditions.

Next, we aimed to assess whether functional IFNs were actually secreted into the culture medium by cells treated with mafosfamide. To do so, CM^mafo^ or CM^veh^ obtained from MCF7 cells as described above were used to stimulate HEK293 cells transiently transfected with luciferase reporter gene constructs driven by the IFN-stimulated response element (ISRE) promoter (see Material and Methods). As shown in Figure 2K, the ISRE reporter construct could be activated by CM^mafo^ compared to CM^veh^. This effect was specific as it was significantly reduced by addition of the JAK inhibitor AG490 (Figure 2K) as well as by expression of a siRNA directed against STAT1 (Figure 2L).

Together, these data strongly suggest that breast cancer cells treated with mafosfamide secrete functional cytokines able to trigger paracrine STAT1 signaling, among which type I IFNs appear to be relevant candidates.

### IFNAR1 and STAT1 silencing effects the genotoxic stress-induced molecular signature

**Figure 3:**
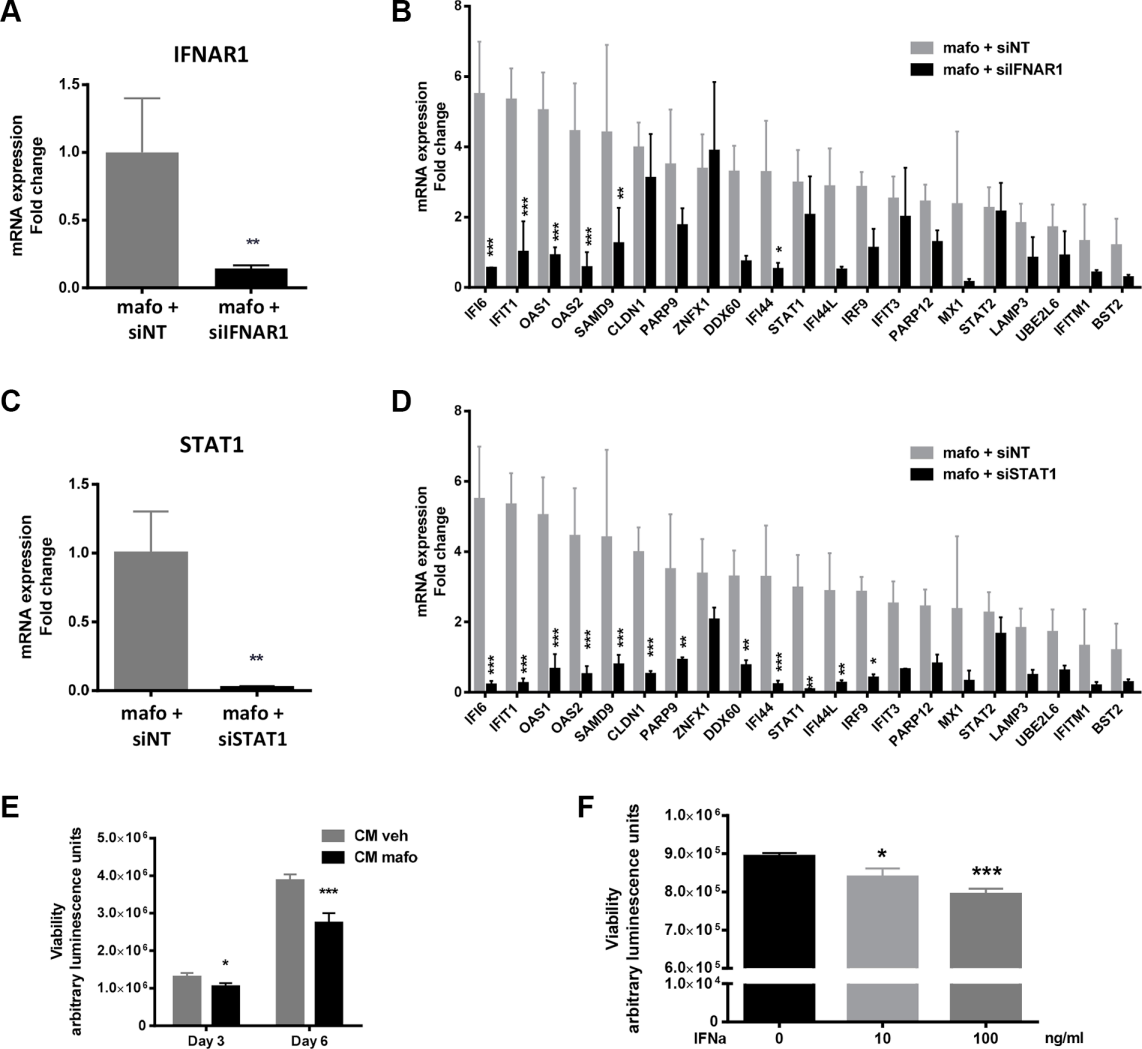
Silencing IFNAR1 or STAT1 gene expression in MCF7 cells impacts on expression of the IFN fingerprint and on cell proliferation (**A**, **B**) qRT-PCR showing the efficacy of IFNAR1 silencing using siIFNAR1 relative to siNT in MCF7 cells treated for 6 days (144 h) with mafosfamide (*n* = 3, mean ± SD, *t* test) (A) and its resulting effect on the expression of the IFN/STAT1 signature (B) (*n* = 3, mean ± SD, two-way ANOVA and post-hoc Sidak's multiple comparison test). (**C**, **D**) The same experiment as in panels A and B is shown for STAT1 silencing. (**E**, **F**) MCF7 viability (Cell Titer-Glo luminescent assay) was measured after incubation in CM^veh^ versus CM^mafo^ for 3 or 6 days (E) or in culture medium containing various doses of purified IFNα (F). In all panels results are expressed as mean ± SD, *n* = 3 independent experiments in triplicate per condition (two-way (E) or one-way (F) ANOVA and post-hoc Sidak's multiple comparison test).

Type I IFNs act on the common receptor, IFNAR, which is composed of two subunits, IFNAR1 and IFNAR2. Both are expressed in MCF7 cells [33]. Former studies showed that IFNAR1 silencing was sufficient to abolish type I IFN signaling [33], therefore we used this strategy to further assess the involvement of paracrine type I IFN signaling. As shown in Figure 3A, a siRNA directed against IFNAR1 markedly decreased its expression compared to a non-targeted siRNA (siNT). Using the latter as a control, IFNAR1 silencing strongly counteracted the ability of MCF7 to express the IFN/STAT1 signature in response to mafosfamide treatment (Figure 3B). Specifically, the induction of 6 out of the 21 genes analyzed (IFI6, IFIT1, OAS1, OAS2, SAMD9, IFI44) was significantly altered, sometimes beyond their level in mafosfamide-free condition. Ten other genes were also decreased (DDX60, IFI44L, PARP9, MX1, IRF9, PARP12, LAMP3, UBE2L6, IFITM1, BST2) but this did not reach statistical significance probably due to heterogeneous levels in the mafosfamide/siNT condition. Finally, the expression level of five genes (ZNFX1, CLDN1, ITIF3, STAT1, STAT2) was not affected. These results confer a key role to IFNAR1, and by extension to type I IFNs, in the induction of the IFN/STAT1 signature observed in MCF7 in response to genotoxic treatment.

In agreement, STAT1 silencing (Figure 3C) produced very similar effect and was even more efficient since CLDN1, PARP9, STAT1, IRF9 and IFIT3 were now significantly down-regulated as compared to the mafosfamide/siNT condition (Figure 3D). This higher efficiency could reflect the stronger inhibition of STAT1 (97.4 ± 1%) vs IFNAR1 (85.7 ± 2.3%) expression. Alternatively, since many cytokines other than IFNs are also able to activate the STAT1 pathway [34], other ligands could also participate in the response observed (e.g. type II IFNs, Interleukin (IL)-6, IL-8, etc.). To address this hypothesis, we performed a cytokine array involving several of these cytokines (Supplementary Figure S4A and S4B). None of them could be detected after 72 h, 96 h (not shown) or 144 h (Supplementary Figure S4) mafosfamide treatment, further supporting IFNs as the major trigger of STAT1 fingerprint downstream of genotoxic stress. Taken together, these data indicate that the type I IFNs/IFNAR1/STAT1 pathway plays a critical role in mediating ISG upregulation in breast cancer cells in response to genotoxic treatment.

Finally, we aimed to address whether the activation of this pathway in response to mafosfamide treatment could affect cell viability. Conditioned media generated as described above were added to naive MCF7 cells and cell viability was measured 3 and 6 days later (Figure 3E). CM^mafo^ (containing secreted cytokines) significantly reduced cell viability compared to CM^veh^, which was reminiscent of the widely described anti-proliferative properties of IFNs [35]. Accordingly, the effect of CM^mafo^ could be mimicked by the addition of purified type I IFNs (Figure 3F). These data suggest that the expression of STAT1 pathway after genotoxic treatment via the production of type I IFNs plays a key role in controlling the cancer cell response.

### The STING pathway is triggered by chemotherapy and contributes to type I IFN production

**Figure 4:**
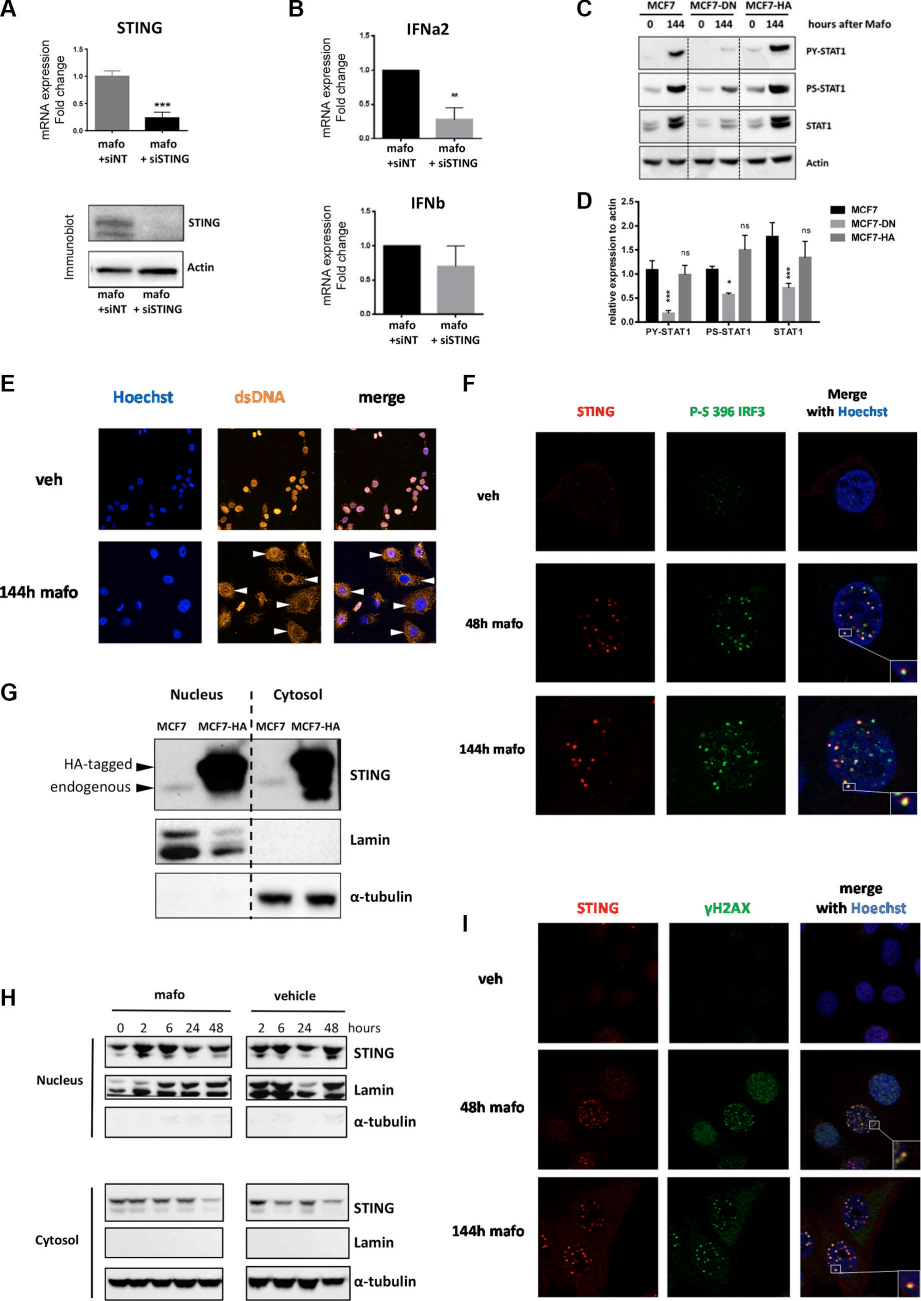
STING pathway is activated and forms nuclear foci in MCF7 cells treated by mafosfamide (**A**) The efficacy of STING silencing using siSTING relative to siNT in MCF7 cells treated for 6 days with mafosfamide was assessed by qRT-PCR (top panel, *n* = 3, mean ± SD, *t* test) and western blotting (bottom panel). (**B**) Expression of IFNα and IFNβ (qRT-PCR) in cells treated for 6 days with mafosfamide in the presence of siNT versus siSTING (mean ± SD, *n* = 3 independent experiments in triplicate, two-way ANOVA and post-hoc Bonferroni's multiple comparison test). (**C**–**D**) Western blotting analysis (C) of P-STAT1^Y701^, P-STAT1^S727^ and total STAT1 after mafosfamide treatment (144 h) of parental MCF7 cells or MCF7 stably expressing a dominant-negative isoform of STING (MCF7-DN) or HA-tagged STING (MCF7-HA); (D) quantifications of the three targets normalized to actin levels are shown for the mafosfamide condition (*n* = 3, mean ± SD, two-way ANOVA and post-hoc Sidak's multiple comparison test). (**E**) Immunofluorescence staining of ss/dsDNA breaks in the cytoplasm (white arrowheads) after 6 day (144 h) treatment of cells with vehicle or mafosfamide. (**F**) Co-immunostaining of STING (red) with P-IRF3^S396^ (green) in cells treated with vehicle or mafosfamide for 48 h or 144 h as indicated. (**G**–**H**) Western blotting analysis of STING in cytosolic and nuclear fractions prepared from untreated MCF7 and MCF7-HA cells (G) and from MCF7 cells treated by mafosfamide or vehicle for the times indicated (H). Lamin and α-tubulin were used as controls of cell fractionation. (**I**) Co-immunostaining of STING (red) with ɣH2AX (green) in cells treated with vehicle or mafosfamide for 48 h or 144 h as indicated. In F and I, nuclei are shown in blue; images shown are representative of 3 independent experiments.

We next aimed to address the potential involvement of STING pathway in the molecular machinery leading to type I IFN expression and signaling in breast cancer cells under genotoxic stress. To that end, we first silenced STING expression. The efficacy of siRNA directed against STING (compared to siNT) was validated by monitoring the down-regulation of STING expression at the mRNA and protein levels (Figure 4A). As shown in Figure 4B, STING knock-down reduced type I IFN upregulation observed in response to treatment, an effect that appeared to be more marked on IFNα than IFNβ. Although this partial effect may reflect residual STING expression (Figure 4A), it may also account for the participation of alternative DNA sensors cooperating with STING such as IFI16 [36], and/or pathways leading to IFN expression in response to genotoxic stress e.g. IRF1 [37] or MYD88 [38], all of which were upregulated in MCF7 and HBCx-19 in response to mafosfamide treatment (Supplementary Figure S5A–S5C). Irrespective of such alternative mechanisms, STING silencing experiments demonstrated the functional involvement of this pathway in mafosfamide-induced IFN expression. To confirm these data, we generated MCF7 cells stably expressing a dominant-negative (DN) isoform of STING [39] or, in control, a HA-tagged version of active STING (Supplementary Figure S5D). As shown in Figure 4C and 4D, expression of STING-DN did not alter baseline expression of total and phosphorylated STAT1. In contrast, it dramatically attenuated the activation of STAT1 signaling in MCF7 cells in response to mafosfamide, as assessed by the decrease in serine-phosphorylated, tyrosine-phosphorylated and total STAT1. Overexpression of STING-HA had no effect compared to parental cells (Figure 4C, 4D), indicating that a certain threshold of STING expression is necessary and sufficient to stimulate the IFN pathway.

Together these results demonstrate that STING pathway is involved in the transcriptional upregulation of type I IFNs triggered by genotoxic treatment of breast cancer cells, resulting in cell-autonomous activation of IFN/STAT1 signaling. This cell-autonomous circuitry then amplifies for several days.

### STING resides in the nucleus, clusters and co-localizes with IRF-3 and γH2AX upon genotoxic stress

As stated in the Introduction, recent studies have shown that ss/dsDNA present in the cytosol of immune cells infected by pathogens could trigger the STING pathway upstream of IFN production. We reasoned that ss/dsDNA release resulting from genotoxic-induced DNA damage in breast cancer cells might have the same effect. Accordingly, we observed that ss/dsDNA breaks accumulated in the cytoplasm after genotoxic treatment of MCF7 cells (white arrowheads on Figure 4E), providing a potential source of STING activators. After sensing DNA the canonical mechanism of STING pathway activation involves the recruitment and activation of TBK1 which in turn phosphorylates serine 396 of IRF-3, a key transcription factor involved in IFN type I transcription [26]. To confirm the activation of this pathway in response to genotoxic stress, we assessed the presence of S^396^-phosphorylated IRF-3. Using immunofluorescence, the latter was detected as foci in the nucleus at 48 h and 144 h timepoints after mafosfamide treatment of MCF7 cells (Figure 4F). Strikingly, STING displayed a very similar nuclear pattern after genotoxic stress; in fact, STING nuclear foci (labelled in red) largely overlapped with S^396^-phosphorylated IRF-3 foci (labelled in green). The number of STING foci significantly decreased upon STING silencing, confirming the specificity of the nuclear staining (Supplementary Figure S5E). Of note, the use of unmasking protocol also displayed the expected location of STING outside of the nucleus envelope (Supplementary Figure S5F).

To gain better knowledge on STING localization within cell compartments, we performed cell fractionation experiments. We noticed that in naive MCF7 cells, endogenous STING was present in both the cytosol and the nucleus (lanes 1 and 3 of Figure 4G). Fractionation of MCF7 cells stably overexpressing HA-tagged STING (see above) fully confirmed this result (Figure 4G, lanes 2 and 4). As nuclear STING foci were not detected in basal conditions but accumulated with in 48 h after mafosfamide treatment (Figures 4F and Supplementary Figure S5G), we fractionated MCF7 cells at various time points between 2 h and 48 h after mafosfamide treatment to detect nuclear translocation of STING. As shown in Figure 4H, the comparison of mafosfamide and vehicle-treated cells did not provide obvious evidence for a quantitative enrichment of nuclear STING linked to genotoxic treatment. This analysis nevertheless confirmed the presence of STING in the nucleus of MCF7 cells. Together, these findings suggest that the formation of STING foci might preferentially result from the clustering of STING present in the nucleus before treatment.

As shown above using western blotting (Figure 1C), γH2AX was detected as early as 24/48 h after mafosfamide treatment, i.e. much before the induction of STAT1 pathway could be detected. As shown by immunofluorescence (Figure 4I), accumulation of γH2AX as nuclear foci (green dots) at sites of DNA damage was detected 48 h after treatment and increased in number and staining intensity until 144 h. Strikingly, merging immunofluorescence images revealed that γH2AX and STING foci largely overlapped (yellow dots). Together, these experiments suggest that under mafosfamide treatment, STING clusters in the nucleus and co-localizes with activated IRF-3 and γH2AX at DNA breaks.

### Cell intrinsic IFN signaling reduces the genotoxic effects, its inhibition amplify them

**Figure 5:**
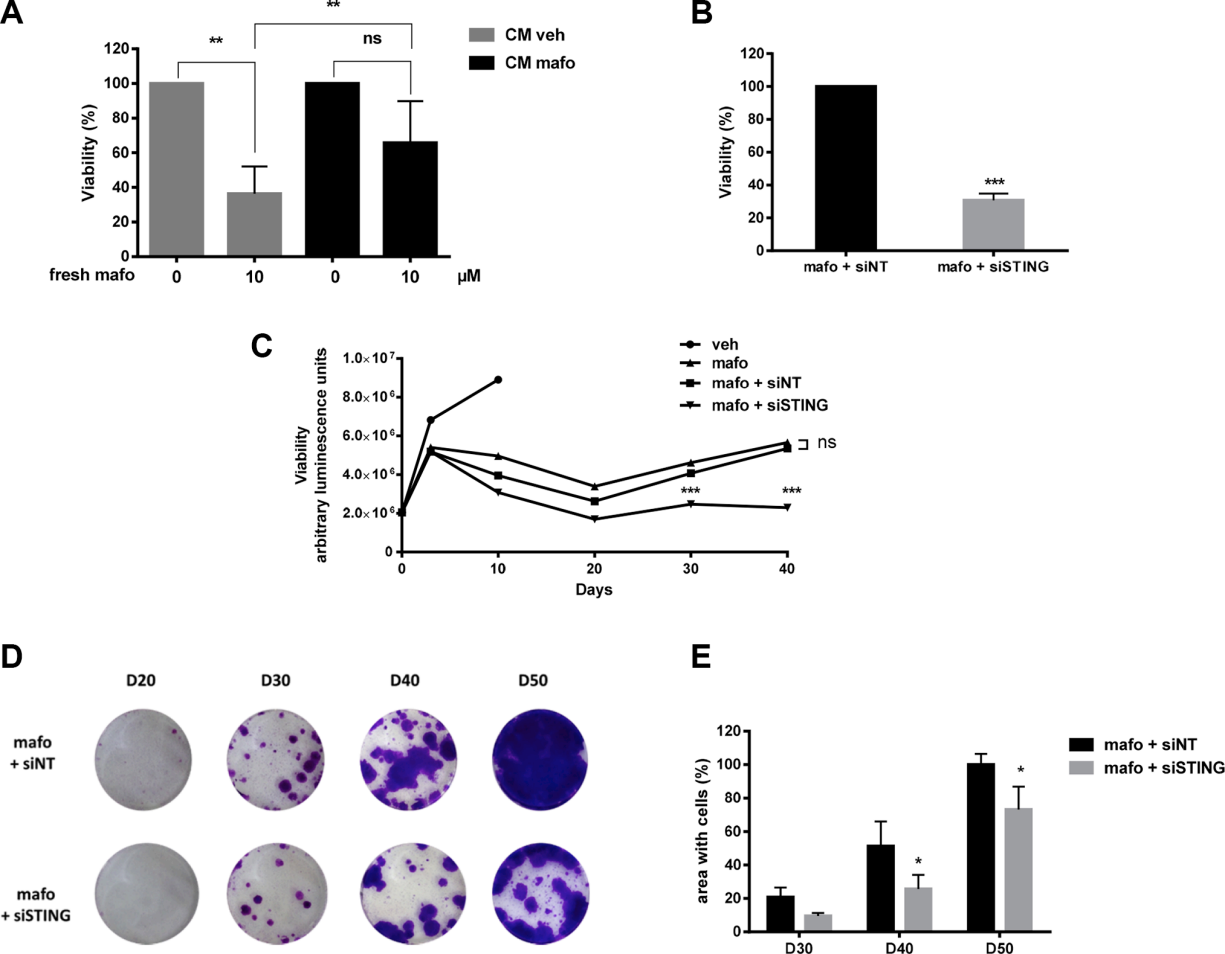
STING silencing potentiates the efficacy of genotoxic treatment of MCF7 cells (**A**) MCF7 cells cultured in CM^veh^ or CM^mafo^ (see Supplementary Figure S3) were treated with mafosfamide then cell viability was assessed after 6 days using CellTiter-Glo luminescent assay. Results are expressed relative to the condition without mafosfamide (mean ± SD, *n* = 3 independent experiments per condition, two-way ANOVA and post-hoc Sidak's multiple comparison test). (**B**) The effect of mafosfamide combined with siSTING or siNT was assessed by CellTiter-Glo luminescent assay 6 days after mafosfamide treatment. Results were relative to mafosfamide with siNT (mean ± SD, *n* = 3 independent experiment per condition, *t* test). (**C**) The effect of mafosfamide on cell viability over 40 days in the presence or the absence of siNT or siSTING was assessed by CellTiter-Glo luminescent assay (two-way ANOVA and post-hoc Tukey's multiple comparison test). (**D**, **E**) Twenty to fifty days after mafosfamide treatment combined with siSTING or siNT the colonies appearing from resistant cells were stained with crystal violet (D) and quantified (E) using ImageJ (mean ± SD, *n* = 3 at per condition, two-way ANOVA and post-hoc Sidak's multiple comparison test).

While CM^mafo^ (containing IFNs and cytokines) reduced cancer cell proliferation (Figure 3E), MCF7 cells cultured for 6 days in CM^mafo^ appeared to be more resistant to mafosfamide treatment as compared to cells cultured in CM^veh^ (Figure 5A). This suggested that priming breast cancer cells with IFNs/cytokines may contribute to confer an increased survival potential to further genotoxic stress. In order to address this hypothesis, we silenced the expression of STING as the most upstream inducer of the IFN production in response to mafosfamide. As expected, STING silencing significantly potentiated mafosfamide treatment efficacy as reflected by the marked decrease in cell viability 6 days after treatment (Figure 5B).

After a single mafosfamide treatment (10 μM), some cells survive but enter into a prolonged steady-state/senescence phase followed by re-growth as colonies detectable between days 20 and 30 (Figure 5C–5E). To address whether STING pathway impacted the tumor recurrence phase, we silenced its expression in MCF7 cells 3 days after treatment and followed them up for the next 50 days. Compared to the control condition (siNT), STING silencing potentiated the efficacy of mafosfamide treatment as reflected by delayed appearance of re-growing colonies of surviving cells.

Together these results suggest that cell-autonomous activation of the STING/IFN pathway confers resistance to treatment which ultimately favors tumor cell survival.

### PARP12 as a novel target to reduce breast cancer resistance to genotoxic stress

A recent study suggested that STING agonists may be of clinical benefit to boost the immune system by upregulating IFNβ and thereby contribute to initial tumor regression [28]. Based on this rationale, we reasoned that STING might be a tricky target *in vivo* contributing to opposite effects in tumor cells (Figure 5) *versus* their microenvironment. We therefore searched for potential targets downstream of the STAT1 pathway. To that end, we developed a lab-scale screening approach using a custom library of siRNA targeting the genes of the IFN fingerprint. In addition to the 21 gene listed in Table 1, a dozen of candidates selected from the transcriptomic analysis of residual PDXs [2] or from the literature (e.g. IFI27, IFIT5, RNAseL, OASL, IFI16, IFIT2, BCL2L2) were added to the analysis based on their potential relevance in the context of interest. Candidate siRNAs were tested individually or by pairs using 96 well plates and cell viability 10 days after mafosfamide treatment was used as the functional readout of gene silencing.

**Figure 6:**
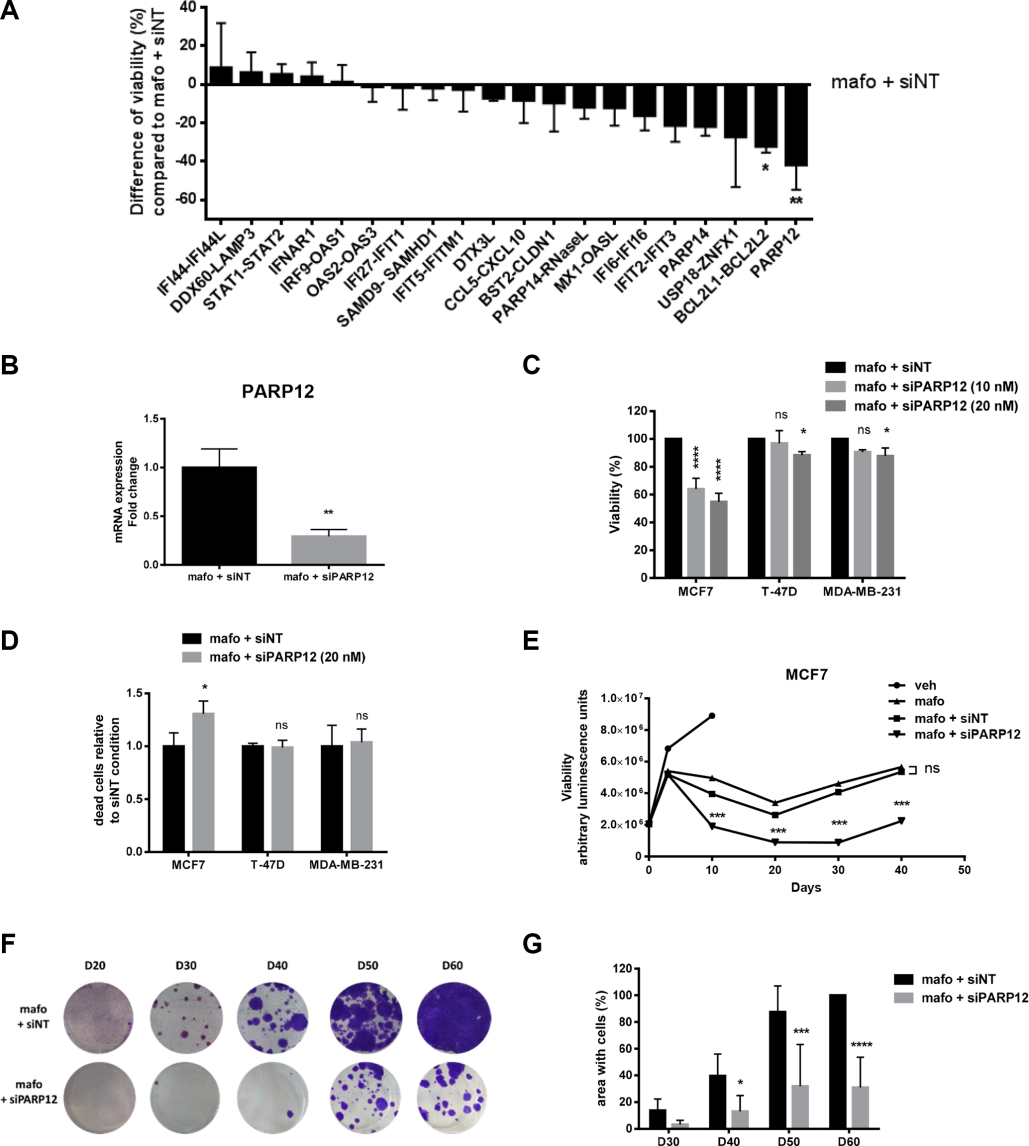
PARP12 silencing potentiates the efficacy of genotoxic treatment of MCF7 cells (**A**) The effect of mafosfamide combined with various siRNAs (tested individually or by pairs) targeting the genes of the IFN/STAT1 fingerprint was assessed by CellTiter-Glo luminescent assay 10 days after mafosfamide treatment of MFC7 cells. Results are relative to mafosfamide with siNT (mean ± SD, *n* = 3 independent experiments per condition, one-way ANOVA post-hoc Dunnett's multiple comparison test). (**B**) The efficacy of PARP12 silencing using siPARP12 relative to siNT in MCF7 cells treated for 10 days with mafosfamide was assessed by qRT-PCR (mean ± SD, *n* = 3 per group, *t* test). (**C**) The effect of mafosfamide combined with siPARP12 or siNT on MCF7, T-47D and MDA-MB-231 cell viability was assessed by CellTiter-Glo luminescent assay 10 days after mafosfamide treatment. Results are relative to mafosfamide with siNT (mean ± SD, *n* = 3 independent experiments per condition, two-way ANOVA and post-hoc Tukey's multiple comparison test). (**D**) The effect of mafosfamide combined with siPARP12 (20 nM) on MCF7, T-47D and MDA-MB-231 cell mortality was evaluated by FACS analysis 10 days after mafosfamide treatment. The results represent the percentage of dead cells compared to mafosfamide with siNT (mean ± SD, *n* = 3 independent experiments per condition, two-way ANOVA and post-hoc Sidak's multiple comparison test). (**E**) The effect of mafosfamide on cell viability over 50 days in the presence or the absence of siNT or siPARP12 was assessed by CellTiter-Glo luminescent assay (two-way ANOVA and post-hoc Tukey's multiple comparison test). (**F**, **G**) Twenty to sixty days after mafosfamide treatment combined with siPARP12 or siNT the colonies appearing from resistant cells were stained with crystal violet (F) and quantified (G) using ImageJ (mean ± SD, *n* = 3 at per condition, two-way ANOVA and post-hoc Sidak's multiple comparison test).

For the majority of the genes silencing resulted in increased mafosfamide-induced cell death compared to siNT, further supporting a role for the IFN fingerprint in conferring cell resistance to treatment (Figure 6A). This preliminary screening did not highlight significant effect of STAT1/STAT2 and IFNAR1 silencing. We focused on the most promising genes and we individually silenced those initially analyzed by pairs (BCL2L1-BCL2L2; USP18-ZNFX1; IFIT2-IFIT3) (not shown). Among all of them, PARP12 appeared to be the gene whose transcriptional inhibition most potently inhibited MCF7 cell viability under genotoxic stress (Figure 6A). To validate this result, we confirmed that the PARP12 siRNA used in the screening experiment efficiently reduced PARP12 expression (Figure 6B) and actually affected MCF7 cell viability when combined to mafosfamide (Figure 6C). A FACS analysis discriminating live from dead cells showed that inhibition of PARP12 combined to mafosfamide induced more cell death than the drug alone, confirming the results of the cell viability assay (Figure 6D). Strikingly, PARP12 silencing had limited impact on T-47D and MDA-MB-231 cell viability and on the percentage of dead cells (FACS analysis) compared to mafosfamide combined to siNT (Figure 6C and 6D), strengthening former observations showing that these two models do not trigger the IFN/STAT1 pathway in response to genotoxic stress. We then performed a colony assay using the MCF7 model in order to evaluate whether PARP12 silencing interfered with tumor survival and growth. A very significant delay (~10 days) in cell re-growth (colony formation) was observed when mafosfamide was combined to siNT *versus* PARP12 siRNA (Figure 6E–6G). Combining STING and PARP12 siRNA did not exhibit additional or synergistic effect (Supplementary Figure S6A, S6B), confirming that these two targets belong to the same functional pathway.

Taken together, these data indicated that breast cancer cell intrinsic IFN signaling induced by genotoxic stress favors resistance to treatment in a PARP12-dependent manner.

## DISCUSSION

This study enlightens several intriguing aspects of response to chemotherapy of cancer cells and their survival after genotoxic treatment. The genotoxic stress triggers IFN production in breast cancer cells which respond to chemotherapy and not in *de novo* resistant tumors, irrespectively of their hormone receptor status and p53 mutated type. Trigger of IFN effect is mediated by the recruitment of STING pathway, providing a mechanism to the recently reported chemotherapy-induced IFN production by cancer cells [2, 11]. Moreover, the cell-intrinsic STING/IFN/STAT1 pathway triggers a typical ISG fingerprint that ultimately contributes to resistance to treatment and favors tumor cell re-growth. Finally this biological process might involves the cooperation of various ISGs among which PARP12 was identified.

The ‘viral mimicry’ concept of cancers has emerged based on the assumption that the mechanisms mediating the innate response to pathogen aggression may also apply to tumor/host relationships [10, 40, 41]. Accordingly, a growing body of evidence argues that tumor cells are under the control of the immune system as highlighted by experimental studies showing that immune activation potentiates the response to genotoxic therapy in various syngeneic or xenograft models [Ref. 10 and references therein, Ref. 42]. Here we pushed this concept one step further by showing that the STING pathway, which is a molecular mechanism typically activated in immune cells in response to pathogen infection (see Introduction), can also be mobilized in cancer cells undergoing genotoxic stress. Activation of the canonical STING pathway in MCF7 cells was assessed by i) IRF-3 serine phosphorylation, ii) relocation of STING (see discussion below), and at the functional level, iii) the abolition of genotoxic-induced type I IFN production and signaling upon STING loss-of-function (gene silencing and expression of STING-DN). This is to our knowledge the first report showing activation of this pathway in neoplastic cells in response to genotoxic treatment.

Various mechanisms of STING activation have been reported [43]. In the immune context, STING can be triggered by cGAMP that is catalyzed by cGAS from dsDNA or ssDNA provided by pathogens [21–23]. A similar mechanism may exist in cancer contexts as we observed that STING pathway activation in MCF7 cells paralleled marked cytoplasmic enrichment in ss/dsDNA following genotoxic stress. The exact nature and origin of this cytosolic DNA are yet to be identified but presumably involve dsDNA or ssDNA leaking from nuclei undergoing DNA damage, in agreement with a recent study showing that STING can be activated by the accumulation of DNA damage due to ATM mutations [27]. Also, it has been shown that cytosolic mitochondrial DNA engaged cGAS and promoted the STING-IRF3 pathway resulting in ISG upregulation [44]. The release of mitochondrial DNA under genotoxic stress may potentially trigger a similar mechanism. Irrespective of the nature and origin of the nucleotides that serve as stimuli, STING activation can involve DNA sensors acting as intermediate between STING and dsDNA, e.g. IFI16 [36]. Of interest, the latter was shown to be upregulated in both IFN-responsive MCF7 and HBCx-19 cell lines after mafosfamide treatment, which may contribute to amplify STING pathway activation through enhancement of cytosolic dsDNA detection. Moreover, cells can have in their endosomes dsDNA coming from dying cells in their environment. In this case, DNA sensing could involve TLR (present in the cytoplasmic or endosomal membranes) via MYD88, an adapter protein shown to promote IFNα production [38]. Of interest, MYD88 expression was also increased in both IFN-responsive cell lines upon genotoxic stress (Supplementary Figure S5C). Various intracellular damage-associated molecular pattern (DAMPs) could also be involved in STING activation. DAMPs include signals (e.g. ssDNA, dsDNA, ssRNA, or dsRNA) that dying cells use to alert the immune system [10]. Further studies are obviously needed to elucidate the DNA sensing circuitry that is mobilized upstream of STING pathway in neoplastic cells undergoing genotoxic stress.

STING has been described to localize in the endoplasmic reticulum, the Golgi apparatus, perinuclear and autophagic vesicles [18, 24, 45]. Here, we show that STING can also be present in the nucleus. Whether this is a specificity of (breast) cancer cells or a more general event will have to be addressed in future studies. Of note, similar observations were made using cells expressing ectopic STING-HA which is frequently used in the field due to technical issues for detecting endogenous STING using commercial antibodies. Intriguingly, STING was already present in the nucleus of MCF7 cells before treatment. While STING nuclear foci peaked within 48 h of treatment, cell fractionation experiments over the same timeline showed no concomitant enrichment of nuclear STING content compared to vehicle-treated cells (Figure 4H). This argues against massive cytoplasmic-nuclear translocation as a major mechanism underlying STING foci formation. We may therefore speculate that STING foci result, at least in part, from the clustering of STING already located within the nucleus prior to treatment. In-depth investigations are needed to assess this hypothesis, as well as to elucidate the molecular mechanisms involved in nuclear STING activation and the actual consequences of STING clustering in the nucleus. In this respect, we noticed that nuclear STING partly co-localized with the phosphorylated histone ɣH2AX, suggesting a new role of STING in response to DNA damage. Although nuclear localization of STING had not been described before, its potential role in chromatin compaction in concert with other histones to potentiate immune signaling has been suggested [46]. IFI16, another DNA sensor commonly found in the cytosol, has been reported to re-localize into the nucleus after DNA or virus stimulation [47] and to associate with BRCA1 during DNA damage repair [48]. Furthermore, Mre11, a well-known protein involved in the MRN complex for double strand breaks DNA repairs in the nucleus, has also been reported to sense DNA in the cytosol [49]. Thus, against the early dogma that the nucleus is protected from DNA detection by DNA sensors [for a review, 43], our observation add another example suggesting that some DNA sensors may be able to sense DNA both in the cytosol and in the nucleus. Elucidating the functional consequences of STING subcellular localization with the DNA damage marker ɣH2AX in breast cancer cells undergoing genotoxic stress deserves further investigations.

Inhibition of STING expression/activity unambiguously showed that STING contributed to chemotherapy-triggered type I IFN production (Figure 4B) and signaling (Figure 4C) in MCF7 cells. Moreover, direct inhibition of downstream IFNAR1/STAT1 pathway identified the latter as a major regulator of the IFN fingerprint (Figure 3). We noticed that the effect of STING knockdown was more marked on IFNα than IFNβ. This may reflect the concomitant upregulation of IRF1 upon genotoxic stress (Supplementary Figure S5B) as this transcriptional regulator was shown to induce IFNβ expression [37]. According to the higher efficiency of STAT1 *versus* IFNAR1 silencing to abolish the IFN/STAT1 fingerprint we cannot exclude that other cytokines present in the chemotherapy-induced MCF7 secretome may also participate in STAT1 activation, though at a lower level than type I IFNs. There are in fact many STAT1 activators [34] of which only a limited set was covered by the cytokine array that we used. Of note, we failed to detect IL-6 and IL-8 which were shown to be upregulated in radioresistant cancer cells expressing the IRDS signature [50], suggesting that distinct mechanisms may be engaged. Other candidate ligands may involve PDGF, EGF, VEGF, GH, or other interleukins such as IL-3, IL-15 or IL-22 [34, 51].

Traditional chemotherapies require active cycling cells to trigger cell death [52]. Cells that are quiescent or slowly cycling are therefore less likely to be responsive to these drugs [53]. After short-term chemotherapy exposure, it has been shown that cancer cells enrich in slow cycling, dormant, chemo-resistant cell sub-populations that can resume growth after drug withdraw [54]. Even cell lines established and grown *in vitro* for decades were shown to contain such slow cycling cells [55]. In our experiments, genotoxic treatment markedly disturbed the progression through the cell cycle as confirmed by dramatic reduction of Ki-67 positive cells (Supplementary Figure S1). However, 20 days after treatment, some cells which survived treatment were able to reenter the cell cycle and form growing colonies. We show here that the chemotherapy-induced MCF7 secretome acts as a survival stimulus reducing cell death (Figure 5A). Remarkably, this effect was blunted by STING down-regulation (Figure 5B–5E), suggesting that the STING/IFN/STAT1 pathway acts as a cell-intrinsic mechanism of malignant cell survival and re-growth. This finding is in agreement with former reports suggesting that STAT1 over-signaling in cancer cells confers resistance to DNA-damaging agents [12–16]. However, in our cell system, not such an effect on cell viability was observed upon silencing of IFNAR1 (Figure 6A). This unexpected result could reflect a role of other cytokines as mentioned above. Alternatively, it may reflect that some genes among the most efficient to reduce cell viability (e.g. PARP12, ZNFX1, IFIT3; see Figure 6A) were not significantly affected by IFNAR1 silencing (see Figure 3B). Finally, although we clearly showed that STING loss-of-function abrogated cell-autonomous IFN signaling, STING may also contribute to maintain the expression of some ISGs via IFN-independent mechanisms [56]. Further investigations are needed to provide convincing evidence for such a mechanism in our cell model, which may help better understand why MCF7 cells die after STING inhibition.

In their elegant work, Sistigu *et al* used type I IFN signaling-deficient (IFNAR1^−/−^) tumors allografted in syngeneic host mice to demonstrate that malignant cell-autonomous IFN signaling promoted anthracycline efficacy by inducing immune responses possibly mediated by CXCL10 [11]. The results we here provide integrate well these findings that warn on the protective role that some cytokines secreted by tumor cells subjected to genotoxic injury may exert on the tumor itself by stimulating the immune system. Accordingly, exogenous supply of type I IFN [11] or induction of endogenous IFN production using STING agonists [28] have been proposed as promising strategies to potentiate antitumor responses. Although STING agonists were shown to promote regression of established tumors and distant metastases in experimental models [28], we are not aware that such compounds have been tested in combination with chemotherapy. From our observations involving *in vitro* and immune cell-free cancer models, we anticipate that IFN enrichment in the tumor microenvironment might lead to paradoxical effects combining antitumor (immune cells) and resistance (neoplastic cells) properties. Furthermore, the benefit of attracting more immune cells to fight tumor cells should be carefully balanced with the perverse effects of constitutive inflammation also shown to promote resistance to treatment [57]. In this versatile context, the identification of individual ISGs contributing to tumor cell resistance downstream of the STING/IFN/STAT1 pathway deserved to be investigated further. Furthermore, as the IFN fingerprint induced by genotoxic stress gathers proteins with very different and potentially opposite functions, this strengthened the likelihood that targeted inhibition of individual genes specifically in tumor cells may be an efficient strategy to potentiate chemotherapy.

Screening the IFN fingerprint identified PARP12 as the most potent contributor to resistance to treatment. Of interest, PARP12 was not identified among IRDS target genes (Table 1). Together with PARP9 (Table 1) and PARP14 [2] also found in the IFN fingerprint, PARP12 is a member of the large family of ADP-ribosyl transferases and is considered as a mono-ADP-ribose transferase like PARP-3,-6,-7,-8,-10,-11,-14,-15, and -16. While the DNA repair properties of canonical PARP members (PARPs 1 and 2) make them attractive targets for cancer therapy, the actual role of other family members is less well (or not) documented in breast cancer [58, 59]. PARP9 was shown to be a member of the DNA damage response pathway and to be part of a DNA repair complex [60]. Moreover, it has been described to repress the anti-proliferative and pro-apoptotic IFN/STAT1 axis in B-cell lymphoma [61]. PARP14 is involved in genomic stability [62]. Of note, genetic inhibition of PARP9 and PARP14 was shown to increase chemotherapy efficacy in prostate cancer cells [63]. PARP12 is much less documented and was shown to be involved in the control of protein translation and inflammation [58]. This study is the first to suggest its implication in cancer. We may speculate that when combined with mafosfamide, PARP12 inhibition reduces dormant cell survival leading to delayed cancer cell colony regrowth. In agreement, using the Kaplan Meier Plotter database (http://kmplot.com/analysis/), we found that ER-positive breast tumors that overexpressed PARP12 had a worse outcome compared to those expressing low levels of PARP12 (Supplementary Figure S6C). Obviously further studies are required to elucidate its mechanism of action in resistance to treatment.

Finally, this study identified two breast cancer cell lines (MDA-MB-231 and T-47D) that were unable to induce the typical IFN fingerprint in response to genotoxic stress. Both cell lines failed to display the rise in IFN production observed 6–8 days after treatment in the two IFN-responsive cell lines, consistent with the absence of STAT1 activation (Figure 1). Of interest, we noticed that in contrast to IFN-responsive cell lines, both MDA-MB-231 and T-47D harbored mutated p53 (Table 2). A link between enrichment of JAK/STAT-regulated genes after genotoxic stress and p53 status has been reported [32]. A combination of 5-bromo-2′-deoxyuridine (BrdU) and distamycin A (DMA) was capable to induce IFN/STAT1 pathway in cells with WT p53 while in cells with mutated p53, the induction was virtually absent. However, in both cases, cells were shown to undergo senescence. Our findings that the four cell lines entered into senescence after mafosfamide treatment irrespective of IFN/STAT1 signature and type I IFN expression, suggests that the lack of IFN/STAT1 signature in T-47D and MDA-MB-231 might be due to alterations of cell cycling regulation as produced by p53 mutations [64, 65]. This point needs to be explored.

Interferons are well known to exert multiple, complex and often opposite roles on cancerous and non-cancerous cells of the tumor microenvironment [10]. This study adds a level of complexity by providing the rationale for considering genotoxic-induced activation of the STING/IFN/STAT1 pathway in breast cancer cells as a cell-intrinsic mechanism of escape to treatment. This finding is in agreement with our recent *in vivo* studies showing that the IFN fingerprint is still present in residual PDX tumor cells giving rise to tumor recurrence in the context of immunodeficiency [2]. Therefore, we propose that therapeutic promotion of IFN signaling in the tumor microenvironment should take into account this tumor cell-specific effect, and consider the potential benefit of targeting downstream gene products. PARP12 has already been identified as a candidate, deeper investigation of the other genes of the IFN fingerprint is currently in progress.

## MATERIALS AND METHODS

### Cell culture, genotoxic treatment and cell harvesting

MCF7 (ATCC, HTB-22), T-47D (ATCC, HTB-133), MDA-MB-231 (ATCC, HTB-26), HEK293 (ATCC, CRL-1573) cells were grown at 37°C and 5% CO_2_ in Dulbecco's Modified Eagle Medium with nutrient mixture F-12 (DMEM/F12) (Thermo Fisher Scientific) supplemented with fetal bovine serum (FBS) to a final concentration of 10%. MCF7 cells stably overexpressing HA-tagged STING (STING-HA) or a dominant-negative STING isoform [39] were obtained by blasticidin (20 μg/mL) selection of cells transfected with cognate plasmids (pUNO1-hSTING-HA3x or pUNO1-hSTING-MRP, respectively; InvivoGen) and maintained in the same culture conditions including blasticidin addition. HBCx-19 was dissociated with Tumor dissociation kit (human, 130-095-929, Milteny) followed by MACS MicroBead Technology (Milteny) to remove mouse fibroblasts. The primary culture was grown at 37°C and 5% CO_2_ in Advanced DMEM/F12 (Thermo Fisher Scientific) supplemented with FBS to a final concentration of 10%. Mafosfamide (D-17272, Niomech) was added once in cell culture media at T_0_ of each time course experiment. At the end of each experiment, cells were washed in PBS, incubated in trypsin for 5 min/37°C, then collected with complete medium. After centrifugation (500 g, 5 min), cells were washed in PBS and kept at −80°C as dry pellet or lysed in a solution containing 50 mM Tris-HCl pH 7.5, 150 mM NaCl, 1% Triton X-100, 1 mM EDTA, 50 mM HEPES, 1 mM NaF anti-phosphatase, 2 mM NA_3_VO_4_ and proteases inhibitors. For senescence assay, cells were stained according to the manufacturer's instructions (ab65351, Abcam).

### qRT-PCR

Total RNA extraction was performed with NucleoSpin RNA XS (Macherey-Nagel) following the manufacturer's instructions. Total RNA (1 μg from each sample) was then reverse transcribed into cDNA with High Capacity cDNA Reverse Transcription Kit (Applied biosystems). Expression of IFN-related genes was analyzed with SYBR Select Master Mix (Life Technologies). qRT-PCR data were invariably normalized to the averaged expression levels of three housekeeping genes: GAPDH (glyceraldehyde 3-phosphate dehydrogenase), RPL13 (ribosomal protein L13) and HPRT1 (hypoxanthine phosphoribosyltransferase 1).

### Cell fractionation

Cells were washed in PBS, incubated in trypsin for 5 min/37°C then collected with complete medium.

After centrifugation (500 g, 5 min), cells were washed in 900 μl cold PBS and spin again. Cell pellets were resuspended in 200 μl of PTG buffer with a protease inhibitors (10 mM Tris-HCl pH 7.4, 2 mM DTT, 10% glycerol, 1 mM MgCl_2_). After 10 min on ice, 2 μl of NP-40 were added to cell suspension and incubated for 3 min at room temperature under gentle stirring. Then the cells were spin 5 min at 1,200 g at 4°C. The supernatant was recovered and kept at -20°C. The pellet was washed 3 times in 200 μl of PTG buffer and was finally resuspended in classical lysis solution.

### Western blot

Cell lysates were assayed using Micro BCA Protein Assay kit (iNtRON Biotechnology). Thirty μg of total proteins were loaded on 4–12% polyacrylamide NuPAGE Novex Bis-Tris gels (Life Technologies) and electrotransferred onto nitrocellulose membranes. Membranes were blocked in PBS with 0,1% of Tween 20 and 3% BSA (1 h, RT), then incubated 2 h at RT or overnight at 4°C with the primary antibodies: STAT1 p84/p91 (sc-346, Santa Cruz, 1/1,000), phospho-STAT1 (tyr701) (sc-135648, Santa Cruz, 1/1,000), phospho-STAT1 (ser727) (07-714, Cell signaling, 1/1,000), ɣH2AX (DR1017, Millipore, 1/1,000) actin (A2066-.2ML, Sigma, 1/1,000), STING/TMEM173/D2P2F (13647, Cell Signaling, 1/1,000), lamin (2032, Cell signaling, 1/1,000), and alpha-tubulin (T15168, Sigma, 1/1,000). The membranes were then washed three times in PBS-Tween and incubated with secondary antibodies (anti-rabbit HRP, 7074 Cell signaling and anti-mouse HRP, NA931 Ge Healthcare). Membranes were revealed with suitable horseradish peroxidase conjugates (Clarity ECL Western Blotting Substrate, Biorad) and quantified by ImageJ software.

### Immunofluorescence

Cells were grown on sterile Lab-Tek Chamber Slide system (Nalge Nune International). Following mafosfamide treatment, cells were washed twice in PBS, fixed in 4% paraformaldehyde (PFA), then washed in PBS. To unmask epitopes (for STING only, as indicated), cells were incubated for 10 min in 50 mM NH_4_Cl, washed with PBS, then permeabilized in 0.1% triton X-100 for 10 min at RT and washed 3 times in PBS. The cells were then blocked in PBS with 0.1% Tween 20 and 3% BSA 1 h at RT and incubated overnight at 4°C with the primary antibodies: STING/TMEM173 (MAB7169, R&D Systems 1/200), phospho-IRF-3 (ser396) (4947, Cell signaling, 1/50), dsDNA (ab27156, Abcam, 1/100), ɣH2AX (DR1017, Millipore, 1/600). The cells were then washed three times in PBS-Tween and incubated with fluorescent secondary antibodies (anti-rabbit Alexa Fluor 488, A-11070 Thermo Fisher Scientific and anti-mouse Alexa Fluor 594, Thermo Fisher Scientific) and kept in the dark. Finally, cells were washed in PBS-Tween, nucleus stained by Hoechst 33342 (10 min, RT) and washed again in PBS-Tween, then rapidly washed in distilled water and covered with slides with fluorescent mounting medium.

### Transfection of siRNAs

Cells were treated 24 h post-seeding or later (as indicated) with 10 μM mafosfamide or vehicle (water). Using interferin reagent (Polyplus transfection), cells were transfected with the following siRNA (GE Dharmacon): siNonTargeted (ON-TARGET*plus* SMARTpool D-001810-10-05), siSTING (ON-TARGET*plus* SMARTpool L-024333-02-0005), siIFNAR1 (ON-TARGET*plus* SMARTpool L-020209-00-0005), siSTAT1 (ON-TARGET*plus* SMARTpool L-003543-00-0005). Using cherry-pick RNAi libraries of Dharmacon, we screened 35 siRNAs (sequences available upon request) in combination with mafosfamide. In brief, cells were seeded in 96-well-plates and treated with 10 μM mafosfamide 24 h later. Three days after treatment, siRNAs were added, then fresh medium was added to the cell three days after siRNAs addition and the plate were analyzed 10 days after mafosfamide treatment using cell viability test (see below).

### Conditioned medium production

MCF7 or HBCx-19 cell lines were seeded in 6-well plates. Twenty four hours later they were treated for 6 h with 10 μM mafosfamide or vehicle, then media were removed, cells were washed twice with PBS and fresh culture medium (without drug) was added. After 144 or 192 h (as indicated), these conditioned media (CM^veh^ and CM^mafo^ coming from cells treated with vehicule or mafosfamide, respectively) were collected then added (without dilution) on naïve MCF7, HBCx-19 or HEK293 cells (Supplementary Figure S3).

### Cytokine array

Cytokines expressed by MCF7 cells after mafosfamide (or vehicle) treatment were identified using human cytokine array (panel A from R&D systems, Minneapolis, MN, USA). Three hundred μg of total protein lysates were incubated with the membrane according to the manufacturer's instructions. Horseradish peroxidase substrate (R&D systems, Minneapolis, MN, USA) was used to detect protein expression. Arrays were scanned using Geldoc apparatus (Biorad) and optical density measurement was obtained with the Image J software.

### Luciferase assay

Luciferase assay was performed in 96-well plate using the Dual-Luciferase Reporter Assay System (Promega) following the manufacturer's instructions. In brief, adherent HEK293 cells were transiently transfected (lipofectamine, Invivogen) with the ISRE (interferon sensitive responder element) luciferase responder plasmid [66] and the Renilla plasmid used for normalization. For experiments involving STAT1 silencing, siSTAT1 or siNT were transiently transfected using interferin reagent (Polyplus) 24 h before reporter plasmid transfection. Twenty four hours later, cells were stimulated for 24 h with CM^veh^ or CM^mafo^, containing or not AG490 (50 μM) or DMSO (vehicle). The luminescence activity in cell lysates was measured using a Mithras LB940 (Berthold) and data were analyzed using the MicroWin2000 software.

### Cell viability assay

Cell viability assays were performed in 96-well plate using CellTiter-Glo Luminescent cell viability assay reagent (Promega) following the manufacturer's instructions. To determine the total growth inhibition (TGI) concentration of mafosfamide, optical density was determined at day 0 versus days 3 or 6 (as indicated). The luminescence was measured with a Mithras LB940 (Berthold).

### Colony assay

After 20, 30, 40 or 50 days of mafosfamide and siRNAs treatment, the cells were washed, fixed with 4% PFA 30 min at RT then washed again with PBS. Next, cells were stained with crystal violet (0.1% crystal violet, 2% ethanol) for 20 min and washed with water. The plate was dried upside-down. Quantification of the area was performed using ImageJ software.

### Statistics

Statistical analyses were performed using GraphPad Prism software. We used multiple *t-test*, one-way or two-way ANOVA as indicated in the legends to figures. One, two and three symbols correspond to *p value* < 0.05, < 0.01 and < 0.001, respectively.

## SUPPLEMENTARY MATERIALS FIGURES


