# Correction: Intracellular STING inactivation sensitizes breast cancer cells to genotoxic agents

**DOI:** 10.18632/oncotarget.27042

**Published:** 2019-06-25

**Authors:** Julie Gaston, Laura Cheradame, Vanessa Yvonnet, Olivier Deas, Marie-France Poupon, Jean-Gabriel Judde, Stefano Cairo, Vincent Goffin

**Affiliations:** ^1^ Inserm, U1151, Institut Necker Enfants Malades (INEM), University Paris Descartes, Faculty of Medicine, Paris, France; ^2^ XenTech, 4 rue Pierre Fontaine, 91000 Evry, France; ^3^ LTTA Center, Department of Morphology, Surgery and Experimental Medicine, University of Ferrara, Italy

We recently reported that a fraction of the protein pool of the DNA sensor STING (also called TMEM173) was present in nucleus of MCF7 breast cancer cells in basal culture conditions [1]. This conclusion was supported by two experimental approaches involving two different anti-STING antibodies: cell fractionation followed by immunoblot analysis using D2P2F rabbit monoclonal antibody (mAb) #13647 from Cell Signaling (Figure 4G in reference 1) and immunohistochemistry using MAB7169 mouse mAb (IgG_2B_) from R&D Biosystems (Figure 4F and 4I in reference 1). While cell fractionation experiments suggested that genotoxic treatment (mafosfamide 10 μM) of MCF7 cells did not enhance the amount of STING present in the nucleus (Figure 4H in reference 1), immunohistochemical analyses suggested that such a treatment promoted the formation of STING nuclear foci (Figure 4F and 4I in reference 1). The reduction of the number of STING-immunostained foci when STING expression was silenced by RNA interference supported the reliability of this observation (Supplementary Figure 5E in reference 1). In addition, we showed that STING-positive foci largely overlapped with foci immunostained with an anti-γH2AX antibody (Figure 4I in reference 1). γH2AX is a phosphorylated form of H2AX histone and is involved in the first step of the DNA damage response process. It is therefore used as a hallmark of double-strand DNA breaks [2].

Based on that data, we aimed to determine the interactome of nuclear STING to elucidate its potential function in the nucleus. For these experiments, we used a cell line generated from patient-derived breast cancer xenograft (PDX) called HBCx-3 [3] as the expression level of STING in the latter was much higher than in MCF7 cells. Cell fractionation confirmed the presence of STING in the nucleus of these cells as previously observed in MCF7 cells (data not shown). STING was immunoprecipitated from nuclei-enriched fractions of vehicle and mafosfamide-treated HBCx-3 cells using anti-STING MAB7169 antibody. Limited amounts of STING were equally immunoprecipitated from both samples (Figure 1A, lanes 1 and 2) while no STING was detected when an irrelevant IgG was used as negative control (Figure 1A, lane 3). These three samples were then analyzed by mass spectrometry. In both anti-STING immunoprecipitates, no STING peptides were detected. Otherwise, irrespective of genotoxic treatment, the most abundantly enriched peptides in anti-STING immunoprecipitates (*versus* negative control) were identified as belonging to Tumor Protein 53-Binding Protein 1 (TP53BP1, hereafter referred to as 53BP1) (Figure 1B). We confirmed by immunoblot the presence of high amounts of 53BP1 in anti-STING immunoprecipitates (Figure 1A, lanes 1 and 2). Both immunoprecipitation/immunoblot (Figure 1A) and mass spectrometry (Figure 1B) experiments failed to detect 53BP1 in the negative control sample, indicating that its presence was linked to the use of MAB7169 antibody. The possibility that the latter precipitated STING/53BP1 complex could not be totally excluded from these observations, but the weak amount of immunoprecipitated STING (Figure 1A) and its non-detection by mass spectrometry was not in favor of this hypothesis. Instead, these results suggested that anti-STING MAB7169 antibody cross-reacted with 53BP1 and was able to immunoprecipitate it in a STING-independent manner.

To investigate this further, we transiently overexpressed a Flag-STING-HA construct in MCF7 cells then we used various antibodies to immunoprecipitate it. In agreement with the results obtained using HBCx-3 cells (Figure 1A), both Flag-STING-HA and 53BP1 were detected in anti-STING immunoprecipitates using MAB7169 antibody (Figure 1C, lane 1). The reverse was not true as Flag-STING-HA was not detected in immunoprecipitates using anti-53BP1 antibodies (lane 7). Larger amounts of Flag-STING-HA protein were immunoprecipitated using anti-HA (lane 3), anti-Flag (lane 4) and the anti-STING antibody D2P2F used above in immunoblots (lane 5), however, 53BP1 protein failed to be detected (Figure 1C, lanes 3-5). Since these three antibodies map different regions of Flag-STING-HA protein, this result supported that, at least in these experimental conditions, 53BP1 and STING do not interact. Hence, the presence of 53BP1 in anti-STING MAB7169 immunoprecipitates (lane 1) likely resulted from STING-independent (non-specific) immnunoprecipitation. Of note, 10-fold higher dilution of MAB169 antibody (lane 2) used in the immunoprecipitation reduced the non-specific cross-reactivity with 53BP1, but this was at the expense of the efficacy of specific STING immunoprecipitation.

The cross-reactivity of anti-STING MAB7169 antibody with 53BP1 observed in these biochemical experiments raised the possibility that the use of this antibody in other types of experiments may also be misleading. In particular, we aimed to re-evaluate our former observation that genotoxic stress promoted the formation of nuclear STING foci in MCF7 cells, as this data was obtained using MAB7169 antibody [1]. To address the specificity of MAB7169-immunostained nuclear foci, we used MCF7 cells transiently overexpressing Flag-STING-HA (Figure 1D shows one transfected and one non-transfected cell). Consistent with our former report [1], weak and diffuse cytoplasmic immunostaining and intense nuclear foci were detected using MAB7169 antibody (Figure 1D, left). Importantly, while cytoplasmic immunostaining was much stronger in transfected than non-transfected cells, nuclear foci were similarly observed irrespective of Flag-STING-HA expression (Figure 1D). The anti-HA antibody confirmed intense and uniformly spread immunostaining within the cytoplasm of transfected cells only (Figure 1D, middle). Notably, no nuclear foci similar to those obtained with MAB7169 antibody were detected using the anti-HA antibody. As a result, merging HA and MAB7169 immunostaining led to fairly good overlapping with the noticeable exception of nuclear foci, further suggesting that the latter are non-specific. Of note, some staining was observed in a distinct area of the nucleus using the anti-HA antibody (white arrow on Figure 4D), which is currently under investigation.

To address whether MAB7169-immunostained nuclear foci could be 53BP1 foci, we silenced 53BP1 in parental MCF7 cells (same cells as used in reference 1) using a targeted siRNA (si53BP1), then cells were analyzed by immunofluorescence. In the control condition involving a non-targeted siRNA (siNT), we observed an almost perfect overlap of nuclear foci immunostained with anti-STING MAB7169 and anti-53BP1 antibodies (Figure 1E, top panels). Silencing 53BP1 expression markedly reduced the number of nuclear foci detected with anti-53BP1 (Figure 1E, bottom middle panel) but also with anti-STING MAB7169 antibodies (Figure 1E, bottom left panel). Genotoxic treatment of cells induces DNA breaks that trigger the DNA damage response machinery, of which 53BP1 is one of the early component involved [4]. Accordingly, mafosfamide treatment of MFC7 cells markedly increased the formation of 53BP1 nuclear foci at DNA breaks (Figure 1F, top middle panel). These foci almost totally disappeared when 53BP1 was silenced (Figure 1F, bottom middle panel). Again, an almost perfect overlap was obtained using anti-STING MAB7169 antibody (Figure 1F, right panels).

Taken together, these results demonstrate that anti-STING MAB7169 antibody strongly cross-reacts with 53BP1 and that the nuclear foci that we misleadingly interpreted as STING foci using this antibody in immunofluorescence [1] are in fact 53BP1 foci. We failed to identify experimental conditions in which specific STING immunostaining by MAB7169 antibody could be increased at the expense of non-specific cross-reactivity with 53BP1. According to the recommendations of the manufacturer, the anti-STING D2P2F antibody failed to provide satisfactory results in immunofluorescence (data not shown). While we were able to confirm the presence of STING in the nuclear fraction using cell fractionation, its precise localization and potential role in the nucleus is yet to be elucidated.

**Figure 1:**
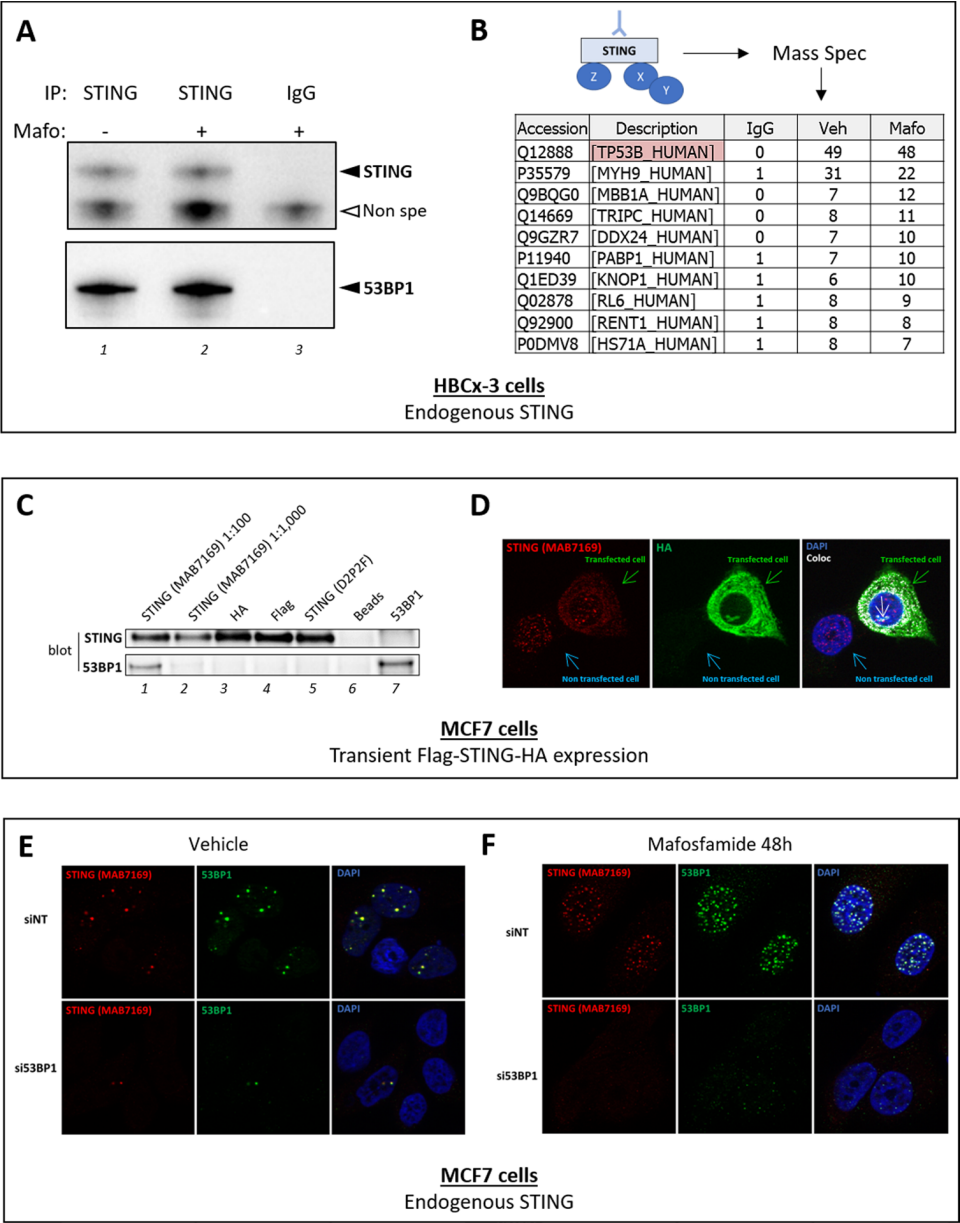
anti-STING mAb MAB7169 antibody cross-reacts with 53BP1. **A-B.** Immunoprecipitation of endogenous STING from nuclear extracts of HBCx-3 breast cancer cells treated or not with mafosfamide (10 μM) using anti-STING MAB7169 mAb (dilution 1:100) or an irrelevant IgG as indicated. In A, immunoprecipitates were analyzed by immunoblot using the D2P2F anti-STING antibody (dilution 1:1,000) and anti-53BP1 antibody (#A300-272A from Bethyl laboratories, dilution 1:1,000). In B, the same immunoprecipitates were analyzed by mass spectrometry and the most significantly detected peptides in anti-STING versus control immunoprecipitates are listed. Peptides corresponding to 53BP1 protein were the most frequently detected, while STING was not in any condition. **C-D.** Immunoprecipitation (C) and immunofluorescence (D) analyses of STING in MCF7 cells transiently expressing a Flag-STING-HA construct. In C, Flag-STING-HA was immunoprecipitated from cell nuclear extracts using antibodies directed against STING (MAB7169 mAb, dilution 1:100/lane 1 and 1:1,000/lane 2, and D2P2F mAb, dilution 1:50/lane 5), HA-Tag (C29F4 from Cell Signaling, dilution 1:50/lane 3) and Flag-Tag (#F1804 from Sigma Aldrich, dilution 1:100/lane 4). Controls involved beads without antibodies (lane 6) or anti-53BP1 antibody (#A300-272A from Bethyl laboratories, dilution 1:100/lane 7). Immunoprecipitates were analyzed by immunoblots using the D2P2F anti-STING antibody and anti-53BP1 antibody as in panel A. In D, Flag-STING-HA expression in MCF7 cells was analyzed using antibodies directed against STING (MAB7169, dilution 1:200) and HA-Tag (C29F4, dilution 1:600). Nuclei were stained with DAPI. One transfected and one non-transfected cells are shown, as indicated. The white arrow in the right panel indicates nuclear staining which differs from the nuclear foci detected with anti-STING MAB7169. Co-localization layout (‘coloc’) was obtained by analysis of merged immunostaining images using ImageJ software. **E-F.** The expression of 53BP1 was silenced (si53BP1) or not (siNT) in parental MCF7 cells, then 24h later cells were treated (F) or not (E) with mafosfamide (10μM). The expression of STING (MAB7169, dilution 1:200) and 53BP1 (#A300-272A from Bethyl laboratories, dilution 1:200) was analyzed 2 days later by immunofluorescence.

Original article: Oncotarget. 2016; 7:77205–77224. 77205-77224 . https://doi.org/10.18632/oncotarget.12858
