# Supplementary material for: Intracellular STING inactivation sensitizes breast cancer cells to genotoxic agents
Source: Oncotarget. 2016 Oct 24;7(47):77205–24. doi: 10.18632/oncotarget.12858 (PMC5363581; doi:10.18632/oncotarget.12858)
Supplement: Supplementary file 1 [file oncotarget-07-77205-s001.pdf]

Intracellular STING inactivation sensitizes breast cancer cells to genotoxic agents

Supplementary Materials

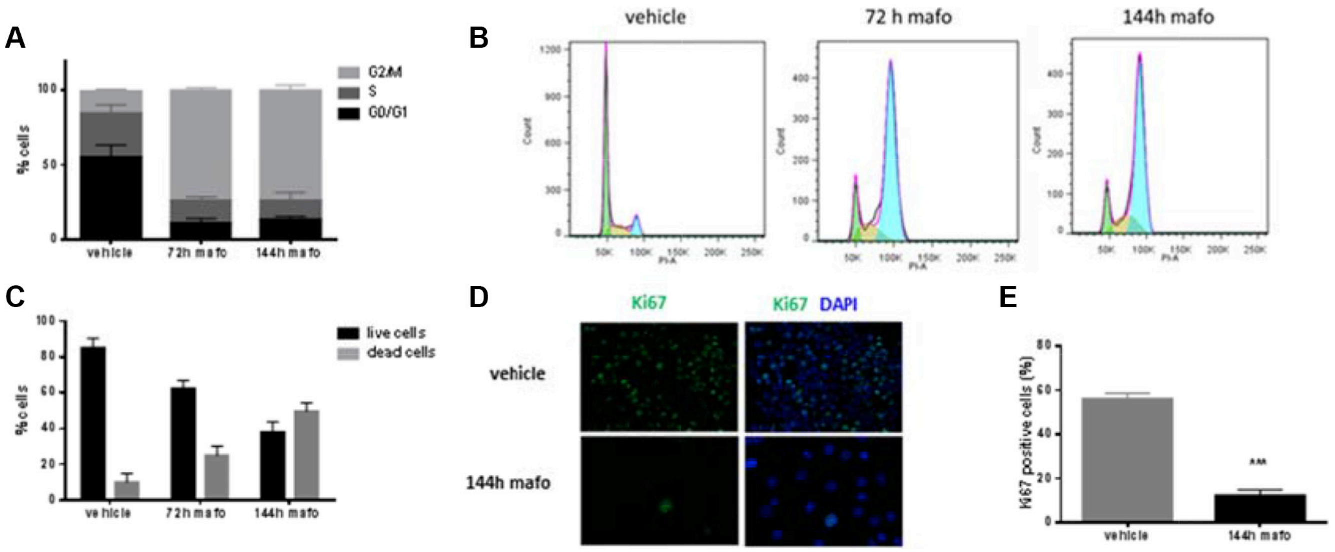

Supplementary Figure S1: Mafosfamide treatment of MCF7 cells alters cell cycle. As assessed by cell cycle analysis, 10 µM mafosfamide treatment blocked cells in G2/M (**A**, **B**). The ratio of live *versus* dead cells was determined by FACS analysis (**C**). Cell proliferation was determined by Ki-67 staining (**D**) and quantified as shown in E ($n = 3$, t test).

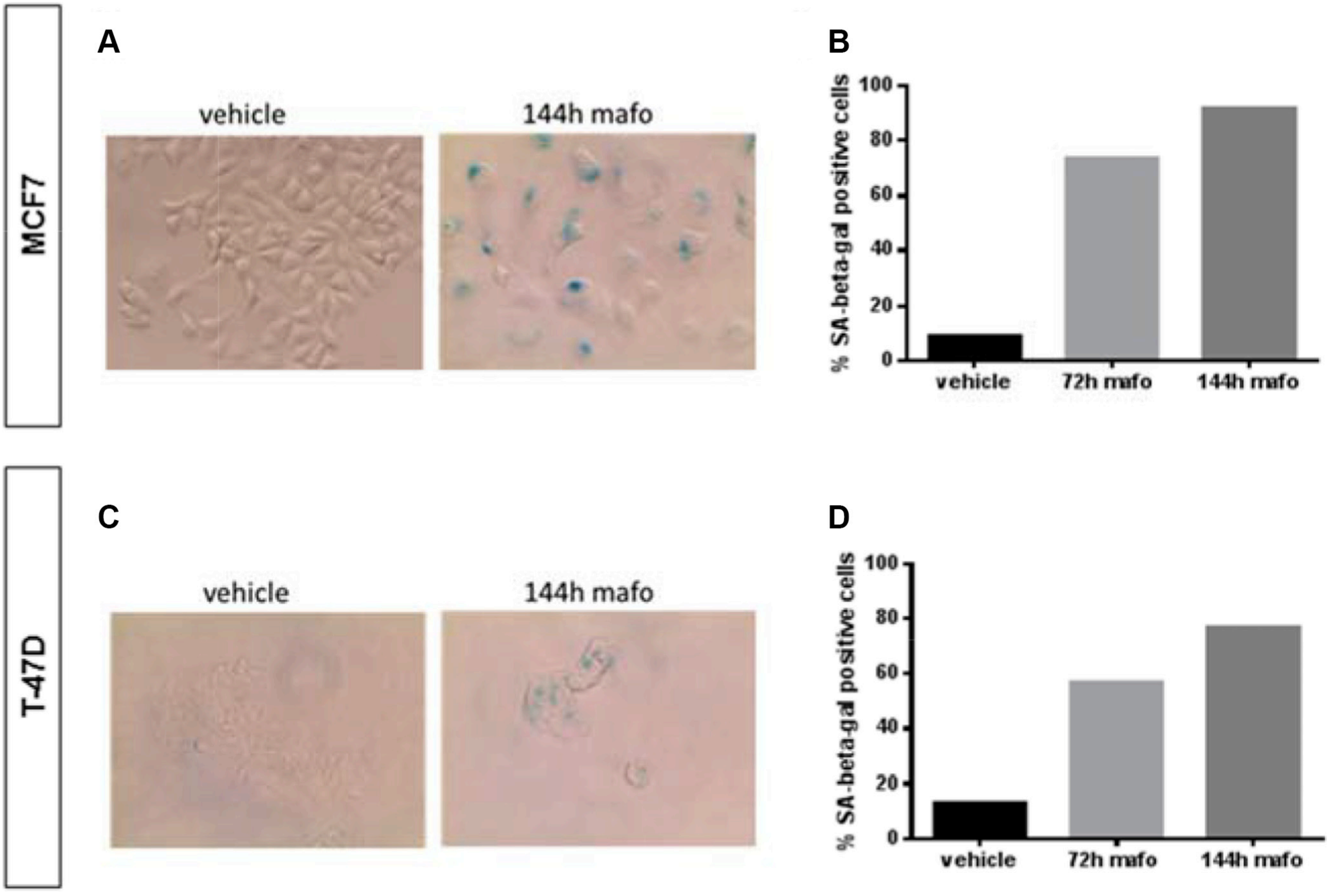

Supplementary Figure S2: Mafosfamide treatment of breast cancer cells induces senescence. (**A**, **C**) The effect of mafosfamide (*versus* vehicle) on cell senescence was assessed 6 days after treatment by senescence-associated β-galactosidase staining in MCF7 (IFN-responsive) and T-47D (IFN-non responsive) cells. (**B**, **D**) The percentage of SA-beta-galactosidase positive cells was quantified from three representative fields of 100 cells each.

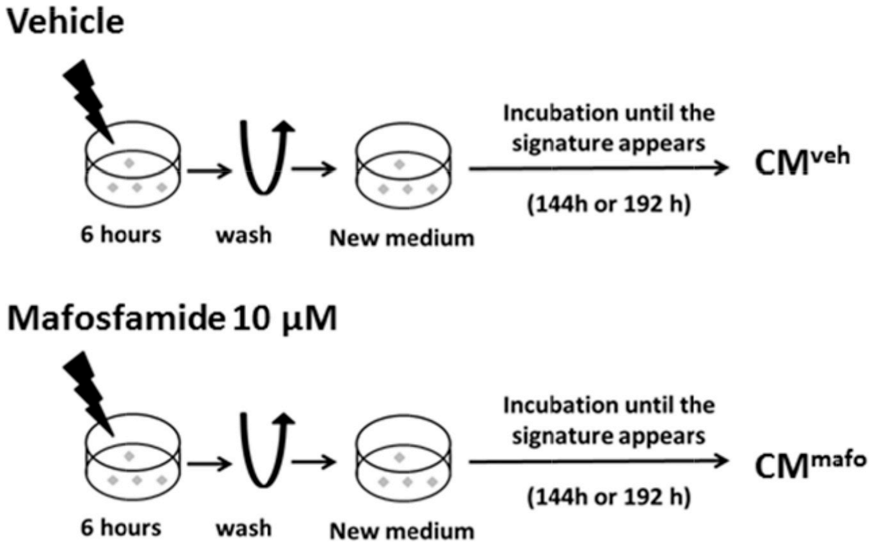

Supplementary Figure S3: Generation of conditioned media. Schematic illustration of the experimental setting to generate conditioned media. 1) Mafosfamide or vehicle were added to the cells during 6 hours. 2) The media were aspirated, cells were washed twice and new medium was added. 3) The medium stayed in contact with cells until the ISGs were overexpressed in the treated cells (144 h for MCF7/192h for HBCx-19). 4) The conditioned media coming from cells treated with vehicle (CM^{veh}) or mafosfamide (CM^{mafo}) were harvested and used for performing cytokine arrays (Supplementary Figure S4) or added onto naïve cells for functional studies (see main text).

A

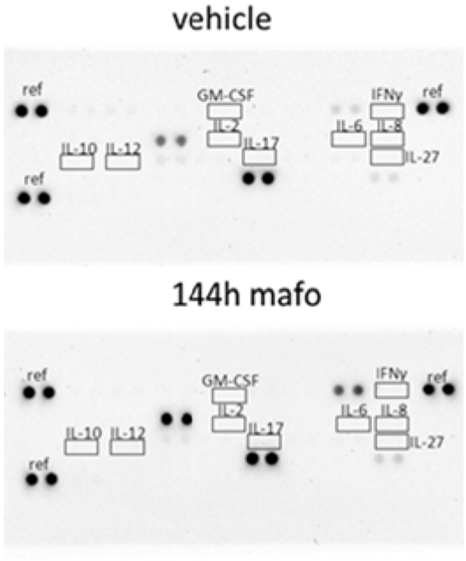

B

STAT1 activators		others	
name	detection	name	detection
GM-CSF	no	C5a	no
IFN-gamma	no	CD40 Ligand/TNFSF5	no
IL-2	no	G-CSF	no
IL-6	no	CXCL1/GRO alpha	no
IL-8	no	CCL1/I-309	no
IL-10	no	ICAM-1= CD54	upregulation
IL-12 p70	no	IL-1 alpha/IL-1F1	no
IL-17	no	IL-1 beta/IL-1F2	no
IL-27	no	IL-1ra/IL-1F3	upregulation
		IL-4	no
		IL-5	no
		IL-13	no
		IL-16	no
		IL-17E	no
		IL-23	no
		IL-32 alpha	no
		CXCL10/IP-10	no
		CXCL11/I-TAC	no
		CCL2/MCP-1	no
		MIF	upregulation
		CCL3/MIP-1 alpha	no
		CCL4/MIP-1 beta	no
		CCL5/RANTES	no
		CXCL12/SDF-1	no
		Serpin E1/PAI-1	upregulation
		TNF-alpha	no
		TREM-1	no

Supplementary Figure S4: Cytokine array analysis of the genotoxic-induced secretome. (**A**) The identification of cytokines secreted by MCF7 treated with mafosfamide versus vehicle was performed 144 h after treatment using cytokine arrays. (**B**) The list of cytokine antibodies spotted onto the cytokine array are discriminated as STAT1 activators (left column) versus other cytokines. None of the STAT1 activators were detected in the genotoxic-induced secretome (see rectangles on the array).

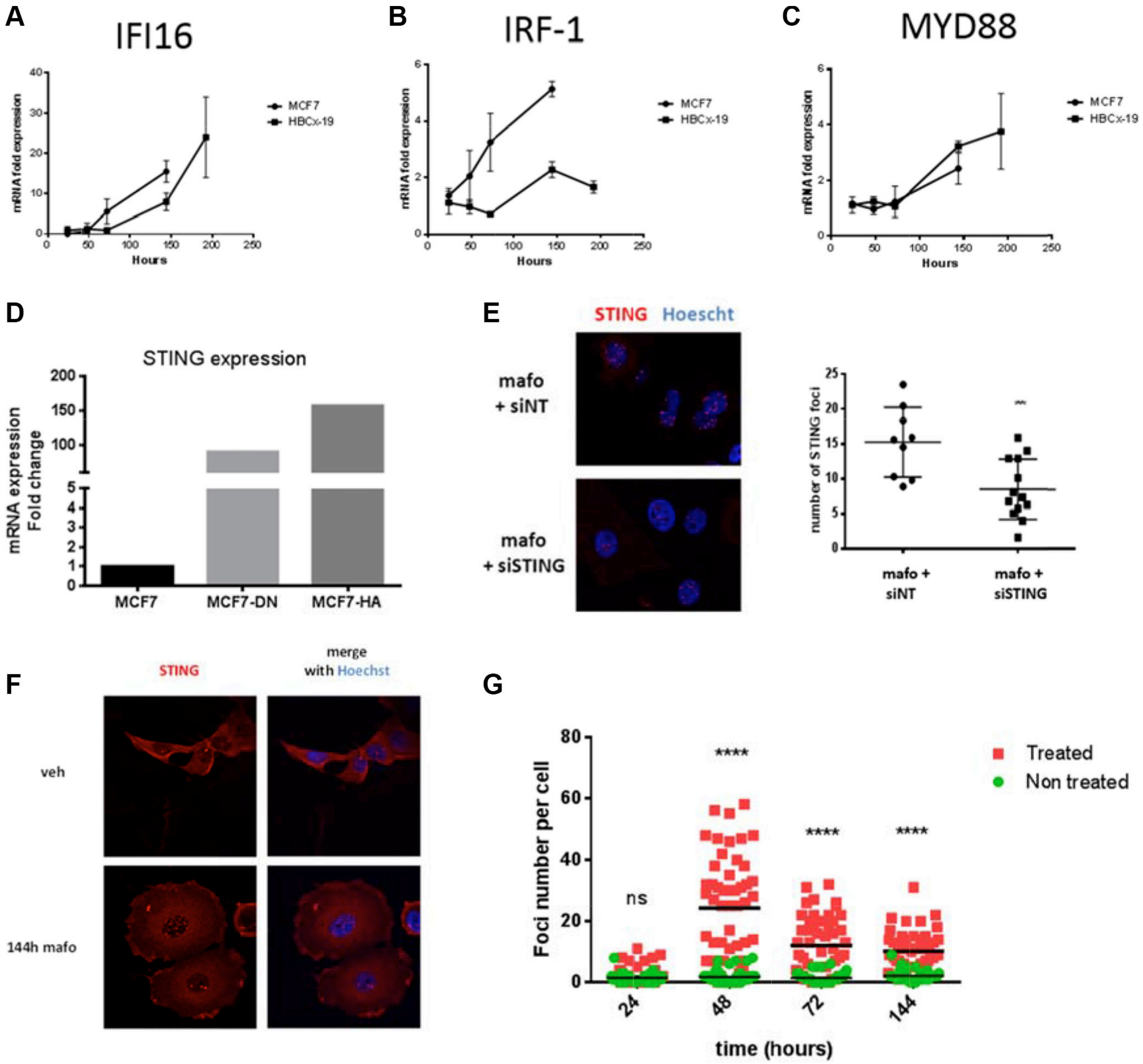

Supplementary Figure S5: Expression of various DNA sensors and subcellular localization of STING upon mafosfamide treatment. (**A**–**C**) qRT-PCR showing the upregulation of IFI16 (A), IRF1 (B) and MYD88 (C) over time in MCF7 and HBCx-19 cells treated with mafosfamide versus vehicle. Results are from three independent experiments. (**D**–**G**) Analysis of STING expression and subcellular localization. (D) qPCR showing overexpression of dominant negative (DN) STING and HA-tagged STING in stably transfected MCF7 cells (called MCF7-DN and MCF7-HA, respectively). (E) Number of nuclear STING foci induced by mafosfamide treatment in MCF7 cells transfected with siSTING versus siNT. (F) Visualization of endogenous STING in the cytoplasm as well as in the nucleus (foci) using epitope unmasking conditions. (G). Time-course quantification of the formation of nuclear STING foci in vehicle (green) versus mafosfamide-treated (red) cells. One typical experiment out of three is shown (50 nuclei counted, two-way ANOVA and post-hoc Sidak's multiple comparison test; ****$p < 0.001$ versus control).

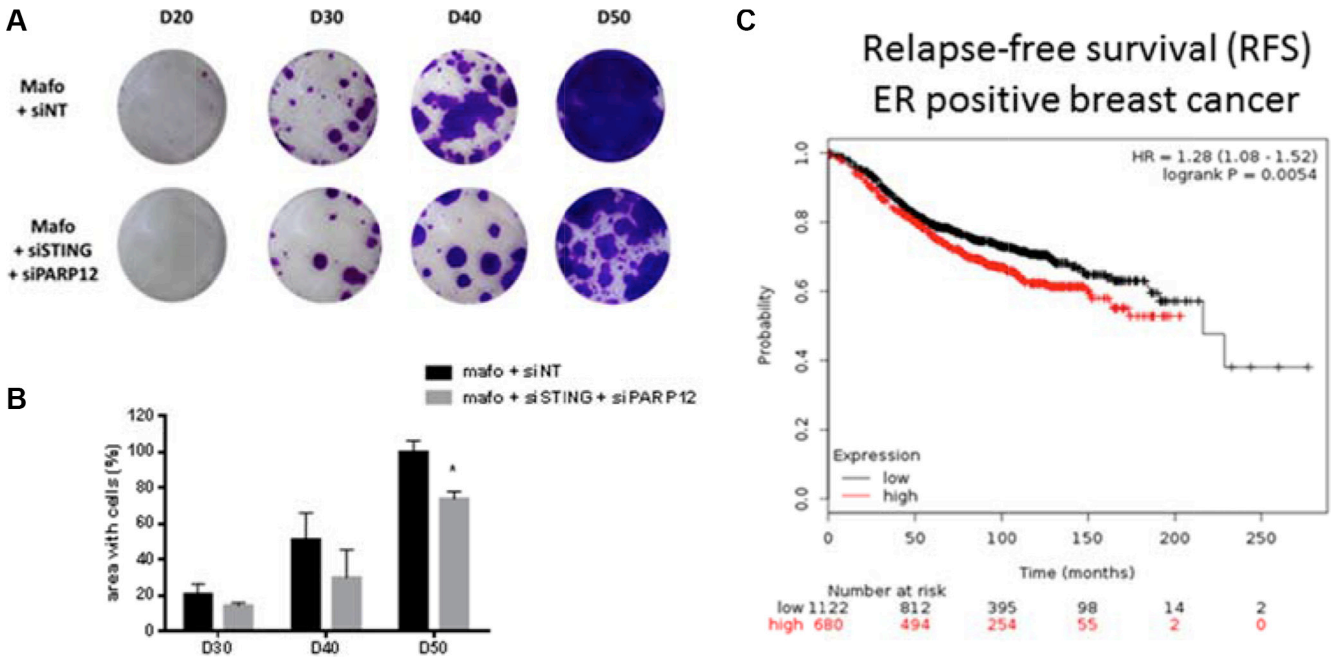

Supplementary Figure S6: (A, B) The combination of siPARP12 and siSTING (here shown *versus* siNT) was not more potent than each single siRNA to potentiate the effect of mafosfamide as assessed by the quantification of colonies appearing from resistant cells stained using crystal violet (see Figures 5 and 6 for additional details). (**C**) Kaplan-Meier plot showing that high expression of PARP12 correlates with lower relapse-free survival in ER+ breast cancers.
